# Dual-Stimulus-Responsive Liquid Marbles with Programmable Motion on Liquid and Hydrogel Surfaces

**DOI:** 10.1021/acs.langmuir.6c03198

**Published:** 2026-07-24

**Authors:** Cansu Goktas, Nihal Aydogan

**Affiliations:** † Department of Chemical Engineering, 37515 Hacettepe University, Beytepe, 06800 Ankara, Turkey

## Abstract

Liquid marbles have attracted a significant amount of attention because of their dynamic programmable nature. In the present study, we propose the conceptualization and implementation of a novel dual-stimulus-responsive liquid marble (LM) platform actuated by near-infrared (NIR) light and magnetic fields and capable of exhibiting controllable locomotion behavior in response to changes in interfacial tension. The integration of external stimuli within a single liquid marble system has been achieved by means of two different micro- and nanoscale functional coating particles and a magnetically responsive core liquid. The liquid marble shell formed through the utilization of hydrophobic polypyrrole particles (SPP), which exhibit high photothermal energy in conjunction with hydrophobic magnetic nanoparticles (F-MNP), which provide magnetic responsiveness. In addition, ferrofluid (FF) has the capacity to respond to an applied magnetic field and is utilized as a core material. The resulting liquid marbles exhibited controllable locomotion under NIR irradiation, magnetic actuation, and combined stimuli through the utilization of near-infrared light-induced Marangoni flows and magnetic field-driven forces. In the presence of external stimuli, these systems generate asymmetric interfacial stress gradients at the liquid–air interface, resulting in the observed controllable locomotion. A systematic investigation was conducted to explore the impact of the particle type, magnetic field, and NIR exposure on the movement behavior of liquid marbles on water and polyacrylamide (PAM) hydrogel surfaces. This work demonstrates the potential of dual-stimulus-responsive liquid marbles as a programmable smart actuation system.

## Graphical abstract

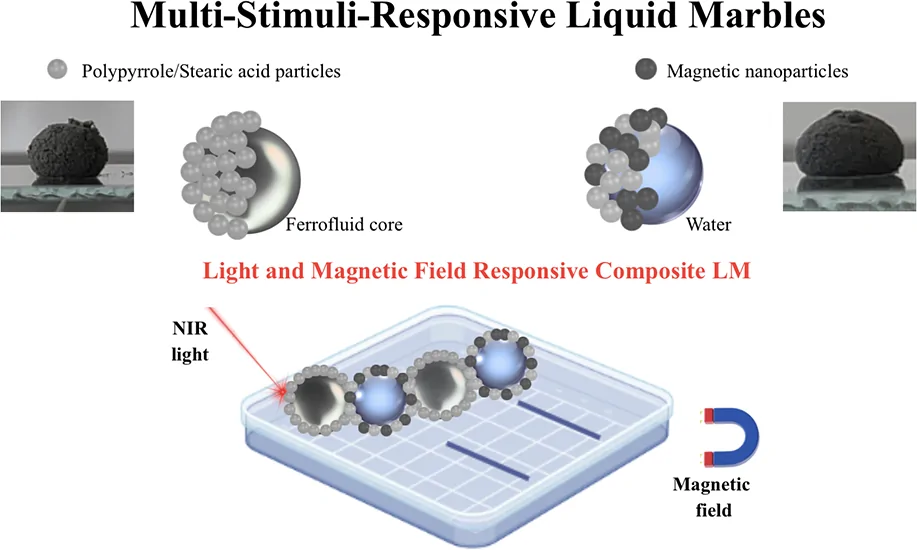

## Introduction

The growing interest in miniature systems has brought liquid marbles (LMs) into greater focus for a range of potential applications, including biochemistry,^1^ microfluidics,^2^ and lab-on-a-chip technology.^3,4^ A liquid marble is defined as a liquid droplet that is coated with micro- or nanoscale solid particles, surrounded by a gas environment.^5^ The nonwettable nature of this structure enables its mobility, and the manipulation of liquid marbles can be achieved through a variety of actuation methods involving a magnetic field,^6–8^ light,^9–11^ gravity,^12^ or a surface tension gradient.^13^ The manipulation ability of liquid marbles has inspired several attempts to prepare movable and multifunctional liquid marbles. The locomotion ability of the LMs in conjunction with their functional both inner party (core) and outer surface makes them a good candidate for different applications.^14^

Miniature soft matter systems have been shown to possess the advantageous capability of responding to a variety of stimuli and performing a range of tasks with high efficiency.^14–18^ The fundamental properties of these systems are determined by the materials used in their construction. The use of materials with additional properties facilitates the fabrication of soft microrobots that possess characteristics such as stimulus responsiveness and mechanical strength.^19–21^ Stimulus-responsive soft materials represent a class of multifunctional, efficient, and intelligent devices that possess the capacity to react to external stimuli, including temperature,^22^ light,^9,23^ ultrasound,^24^ pH,^25^ and magnetic field.^7,26^

The magnetic field has a critical influence on the motion of LMs as an external stimulus. Magnetic liquid marbles, a subclass of LMs composed of magnetic materials, can be fabricated by using two primary methods. The first method uses magnetic nanoparticles in the shell, whereas the second uses a magnetic fluid in the core. Bielas et al. have created hydrogel LMs that have been functionalized with magnetic materials, including magnetosomes, in both liquid and gel structures and exhibited their enhanced responsiveness to external magnetic fields.^27^ Ferrofluids are colloidal liquids that can be used as magnetic liquid cores, wherein their behavior is influenced by the presence of a magnetic field.^28–30^ The initial utilization of ferrofluid within a liquid marble was investigated; polyvinylidene fluoride was employed to coat the ferrofluid droplet, and an external magnet was utilized to manipulate the liquid marble. The resultant ferrofluid liquid marble was shown to achieve a velocity of 25 cm/s.^31^ In a further study, the movement of a ferrofluid marble with alternating attractive and repulsive magnetic forces was demonstrated as a strategy to combine transportation and mixing.^32^ Recent research conducted using a ferrofluid core has demonstrated that the response of a liquid marble to an external magnetic field is determined by the particle type and shell structure. Furthermore, it was demonstrated that ferrofluid-based liquid marbles are capable of inducing internal heating effects in the presence of alternating magnetic fields.^33^ Ferrofluid marbles have been successfully employed as magnetically responsive substances to manipulate biocompatible nonmagnetic liquid marbles through water surface deformation.^34^ Despite an increasing amount of research on ferrofluid liquid marbles, studies combining the literature on ferrofluid core liquid marbles with a ferrofluid core and a photothermally active particle shell remain limited.

In addition to the magnetic field, a significant amount of attention has been drawn to light responsiveness due to its capacity to convert optical energy into mechanical motion. The incorporation of photothermal or photoactive materials into the marble shells of these systems enables directional movement or deformation upon light irradiation, resulting in localized heating. For instance, Fujii et al. fabricated a liquid marble using polypyrrole overlayers and examined its movement when near-infrared laser irradiation was used as an example of light-triggered LMs.^22^

The motion of liquid marbles is strongly influenced by variations in interfacial forces and stress distributions at the interface, as the droplet core is isolated from the surroundings by the coating shell. Additionally, the movement of liquid marbles is determined by the characteristics of the surface.^35,36^ On solid surfaces, rolling and sliding are the predominant mechanisms. The rolling of liquid marbles is determined by adhesion forces that act within the surface contact area.^37^ The motion of liquid marbles can be induced by external stimuli, with gradients in interfacial stress being a driving force.^38^ For example, localized photothermal heating can be produced by light irradiation, leading to surface tension gradients along the interface. The transportation of floating liquid marbles is facilitated by light-induced Marangoni flow. The presence of liquid marbles within a water solution, accompanied by light-responsive particles, results in the generation of photoreversible Marangoni flows. These flows, in turn, facilitate the transportation of liquid marbles away from the light source.^9^ Furthermore, magnetic fields have been shown to induce movement when magnetic particles are incorporated into the shell or liquid core. In such systems, magnetic forces acting upon the particles generate interfacial stresses or body forces, which, in turn, result in the directed motion of the liquid marble.^39^ It is important to improve our current understanding of the motion of liquid marbles.

LMs have emerged as promising soft microactuators and microreactors owing to their unique particle-stabilized interfaces and ability to encapsulate functional liquids.^40^ Previous studies have demonstrated various single-stimulus LM systems, including light-induced thermal actuation using photothermal particles,^21^ magnetic control via magnetite-coated shells,^36^ and ferrofluid-based deformation under external fields.^41^ A previous study has demonstrated synergistic bifunctional liquid marbles by integrating different functional particles in patchwork-like structures to achieve control on super-repellent surfaces.^42^ More recent efforts have attempted a dual-responsive LM configuration, yet these systems typically exhibit limited motion control, rely on free-space actuation without deterministic guidance, or lack an integrated mechanism that couples multiple stimuli within a unified structure. To this end, a comprehensive investigation into diverse stimulus pairs and the movement of liquid marbles on various surfaces is essential. Although photothermal and magnetic liquid marble systems have previously been documented in the literature, to the best of our knowledge, there is an absence of LM platform liquid marble systems that systematically combine photothermal shell design with shell- and core liquid-based magnetic responsiveness for programmable, channel-confined locomotion on both water and soft hydrogel surfaces. This gap highlights the need for a multimodal, hierarchically designed LM system in which nanoscale interfacial engineering directly governs the soft robotic behavior. In this context, our work introduces an LM platform that synergistically merges polypyrrole-based photothermal actuation, magnetic nanoparticle-driven directionality, and ferrofluidic dynamic response, enabling on-demand and dual-stimulus locomotion on water and soft hydrogel surfaces within controlled microarchitectures.

In this study, a liquid marble exhibiting dual-stimulus responsiveness to light and magnetic field is presented, and its potential applications in lab-on-a-chip technology are explored. The integration of photoresponsiveness and magnetic responsiveness into a single liquid marble constitutes a novel material combination. This dual-responsive liquid marble offers precise and remote control of motion and functionality on water and soft hydrogel surfaces. Furthermore, the actuation behavior of the liquid marble is systematically analyzed under light-only, magnetic-only, and combined stimuli to determine the precise and synergistic effects of each stimulus on the marble’s movement. This integrated design offers enhanced controllability of liquid marble locomotion in response to individual and combined stimuli, thereby establishing a foundation for further research on multifunctional liquid marble manipulation through diverse interfaces. For this purpose, magnetic and photoresponsive particles have been prepared, through appropriate surface modification, and through integration of different particle combinations, a dual-stimulus-responsive liquid marble has been created. Furthermore, a ferrofluid liquid marble with a polypyrrole coating has been fabricated, which effectively combines the magnetic responsiveness of ferrofluid and the light-sensitive character of polypyrrole particles. This liquid marble demonstrates responsiveness to both magnetic fields and light, thereby enabling precise control over its movement.

## Experimental Section

### Materials

In the present study, stearic acid, pyrrole, iron­(III) chloride, iron­(III) sulfate, sodium hydroxide, 1*H*,1*H*,2*H*,2*H*-perfluoro-1-decanol (FTOH), tetramethylammonium hydroxide pentahydrate (TMAH), and phenolphthalein were obtained from Sigma-Aldrich. The chemicals utilized in this study were not subjected to any purification process. All solutions were prepared using ultrapure water (UP) with a resistivity of 18.2 MΩ cm (Millipore Sigma, Burlington, MA).

### Synthesis of Surface-Modified Magnetic Nanoparticles

The coprecipitation method was utilized for the synthesis of surface-modified magnetic nanoparticles. Initially, FeCl_3_·6H_2_O and FeSO_4_ salts were dissolved in UP, whereafter they were mixed with a NaOH solution under a N_2_ atmosphere.^43^ The pH was increased to 10 via the gradual addition of a NaOH solution. Following the addition of oleic acid (100 μL), the solution was stirred for 1 h. Thereafter, the solution was heated to 95 °C for the purpose of imparting magnetic properties to the iron hydroxides. The room-temperature solution was then washed thrice with UP and acetone. For the purpose of surface modification, 1*H*,1*H*,2*H*,2*H*-perfluoro-1-decanol (FTOH) was added to the solution and sonicated. The obtained magnetic nanoparticles were then dried in an oven at 40 °C.^7^

### Synthesis of Polypyrrole-Coated Stearic Acid Particles

The preparation/synthesis of polypyrrole (PPy)-coated stearic acid particles was performed through the utilization of a 5 wt % PPy:stearic acid ratio for adequate surface coating. Briefly, pyrrole was added to water (40 mL), and the solution was stirred for 30 min. Then, stearic acid was introduced into the solution, and the mixture was stirred for a further 60 min. FeCl_3_·6H_2_O was utilized as the oxidant and incorporated into the system by dissolution in water. The chemical oxidative polymerization process was conducted over a period of 6 days. The resultant product was subjected to multiple washings via centrifugation and subsequently dried using a freeze-dryer, in preparation for utilization.^22^

### Ferrofluid Synthesis

Ferrofluid synthesis has been adapted from the literature with slight modifications, with the ferrofluid being synthesized via the coprecipitation method. The iron sources utilized were FeCl_3_·6H_2_O and FeCl_2_·4H_2_O, and NaOH was employed to render the solution alkaline. Following the dropwise addition of the NaOH solution, a black color was observed. The agglomeration of the nanoparticle sample was prevented by adding a 25% solution of tetramethylammonium hydroxide pentahydrate. The black liquid was stored at room temperature for further analysis.^44^

### Preparation of Liquid Marbles

LMs were fabricated by means of the rolling method.^5^ Prepared powders were layered on a Petri dish, and a determined volume (10, 20, and 50 μL) of UP or ferrofluid that could be applied was dispensed over the powder using a micropipette. The liquid droplet was rolled over the powder bed for 30 s. The prepared liquid marble was then transferred onto a flat glass substrate or to a channel, and a digital camera was utilized to capture images of the LM.

### Structural Characterizations

Optical microscopy images were taken by using a Leica DMI 4000B instrument to determine the particle size and morphology. Samples were analyzed in suspension at room temperature. Scanning electron microscopy (SEM, Bruker, Zeiss Evo 50) was used for size and geometry measurements. Dynamic light scattering (DLS, ALV-CGS-3 compact goniometer) was used to determine the hydrodynamic radius and size distribution of MNP. In order to identify the atomic groups, present in the molecular structure of the synthesized particles, a Thermo Scientific Nicolet 6700 FTIR instrument was used. The structural characterization of the dried SPP and MNP was examined at wavelengths ranging from 500 to 4000 cm^–1^. In contact angle measurements (Krüss DSA30E drop shape analyzer), 30 μL water droplets were dropped onto compressed particulate dust on the surface, and the contact angle of the liquid droplet resting on the particulate dust was measured. The measurement of the contact angle was conducted through the observation of a sessile drop, at the points of intersection (three-phase contact points) between the drop contour and the baseline. The magnetic properties of F-MNP were characterized at room temperature by using the vibrating sample magnetometer (VSM) option of the Quantum Design Physical Properties Measurement System (PPMS). Electron paramagnetic resonance (EPR) measurements were completed using a Bruker EMX 131 X-band ESR spectrometer (Germany) operating at a frequency of 9.853 GHz and a microwave power of 10.0 mW. The spectra were recorded over a magnetic field range of 0–4500 G.

### Trajectory and Velocity Analysis of LMs

The movement of liquid marbles in micromotor applications was studied in two channels of varying dimensions (11 cm × 6 cm and 35 cm × 7.5 cm) using either pure water or a 1% polyacrylic amide solution. The locomotion of the LMs was recorded with a digital camera using a 1.5 W 808 nm NIR laser (CNI model with PSU-III-LED, MDL-III-808) and 254–480 mT permanent magnets, and speed and trajectory analyses were performed using Tracker video analysis software. The position of each liquid marble was determined using its center as a reference point. The measurement of the total distance traveled was calculated through the calibration of the known channel dimensions. Displacement–time data were obtained for each experiment, and the average velocity was calculated from the change in displacement with time. For each of the experimental conditions, a minimum of five independent measurements were conducted (*n* = 5), and the velocity values are reported as the mean ± standard deviation.

## Results and Discussion

### Preparation and Characterization of Particles

In this study, in order to prepare external stimulus-responsive LMs, stimulus-responsive materials were first prepared. The employment of superparamagnetic nanoparticles within liquid marble systems is predicated on the premise that these systems exhibit a high degree of sensitivity to the magnetic field. In addition, polypyrrole nanoparticles have been used to induce light sensitivity.

### Fabrication and Properties of Magnetic Nanoparticles (F-MNP)

In recent years, the use of superparamagnetic iron oxide particles has been expanded significantly across multiple disciplines, including controlled drug delivery, magnetic storage media, and magnetic resonance imaging.^45^ The utilization of magnetic nanoparticles as coating particles in liquid marbles is significant with regard to their controllability by an external magnetic field. In the present study, the preparation of magnetic nanoparticles was achieved through the coprecipitation of Fe^2+^ and Fe^3+^ ions. It has been demonstrated that bare iron oxide nanoparticles have a tendency to aggregate in aqueous environments. To overcome this limitation, oleic acid was used to coat the particle surfaces. However, to achieve more precise control over the wettability of the particles, a second coating procedure was implemented. The presence of fluorinated chains within the structure has been demonstrated to be a factor that significantly contributes to the reduction of surface energy. This approach facilitates magnetic controllability by preventing aggregation and ensuring the integrity of the liquid marbles through the coating of their surfaces with suitable agents.

The DLS technique was employed to analyze the size distribution and hydrodynamic diameter of the synthesized magnetic nanoparticles shown in Figure 1A, revealing a narrow size distribution and a nearly spherical shape with an average diameter of 51.3 nm and an aspect ratio of 1.1 ± 0.05. It was determined by analyzing five independent samples with 10 measurements for each sample. A FTIR spectrum analysis reveals a distinctive peak at 500–600 cm^–1^, indicative of Fe–O bond stretching vibrations. The Fe_3_O_4_ spectrum exhibits a single absorption peak at a wavelength of 560 cm^–1^. Furthermore, the spectrum demonstrates surface functionalization. Peaks at approximately 2900 cm^–1^ are attributed to CF_2_ and CF_3_ stretching vibrations of 1*H*,1*H*,2*H*,2*H*-perfluoro-1-decanol. The peak at 1300 cm^–1^ is attributed to the C=O stretching vibrations of the carboxyl group of oleic acid (see Figure 1B).^46^ After surface modification, the water contact angle of the coated magnetic powder was measured as 141.6 ± 2.2° (*n* = 35), as shown in Figure 1C, which shows the hydrophobicity of the particles. The contact angle of the liquid marbles can be correlated with the wettability and interfacial energy balance of the particle-coated interface. It is evident that large contact angles are indicative of a strong nonwetting character. This creates a reduced contact area, and it has been shown to decrease frictional resistance, thereby facilitating the mobility of the liquid marbles in response to external stimuli.^47^ This finding indicates the occurrence of successful surface coating. It has been determined that the application of these dual coatings serves to enhance colloidal stability and hydrophobic character. Further magnetic characterization of the F-MNP was conducted using vibrating sample magnetometry (VSM), and the resultant magnetization curve is presented in Figure S3. The VSM results demonstrated typical superparamagnetic characteristics exhibiting negligible coercivity and remanent magnetization at ambient temperature.^48^

**Figure 1:**
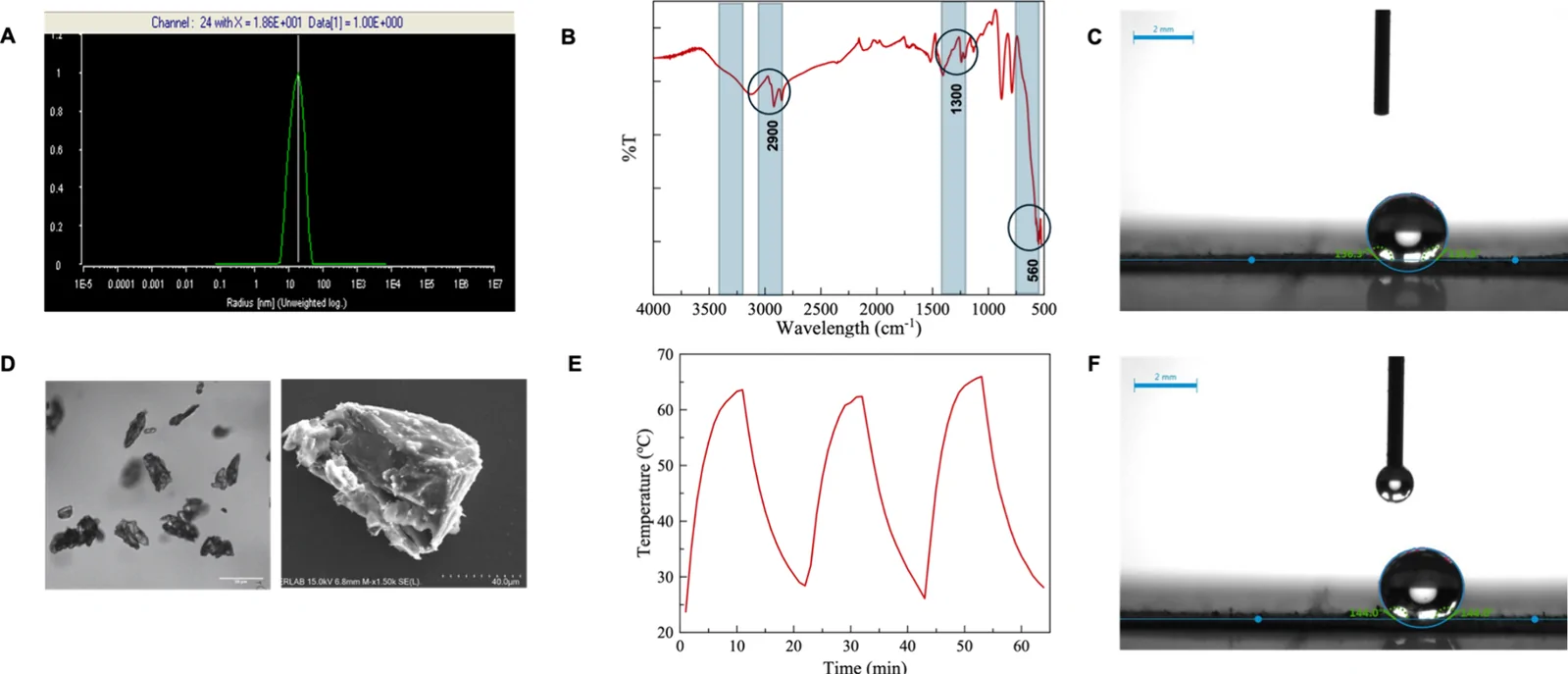
(A) Hydrodynamic radius, (B) Fourier transform infrared (FTIR) spectra, and (C) water contact angle of F-MNP. (D) Optical microscope and SEM images of SPP. (E) Temperature change profile of SPP under an 808 nm NIR laser. (F) Water contact angle of SPP powder.

### Synthesis and Characterization of Stearic Acid-Modified Polypyrrole Particles (SPPs)

The light-responsive motion of liquid marble systems is considered to play an essential role in the development of dual-stimulus liquid marble systems. In this regard, stearic acid-modified polypyrrole particles were synthesized. The morphology and size of the particles were analyzed using an optical microscope and a scanning electron microscope (SEM), respectively. The diameter of the particles was found to be 10.6 ± 2.4 μm, and their length was found to be 17.4 ± 4.5 μm after analysis of 30 particles. Given the asymmetric geometry of the particles, the aspect ratio was calculated to be 1.5 ± 0.06 (see Figure 1D). In order to demonstrate that the polymerization process had been successfully achieved and that PPy had been doped with stearic acid following polymerization, FTIR analysis was performed, and the results are presented in Figure S2. The characteristic peaks at 2916 and 2848 cm^–1^, arising from the asymmetric and symmetric stretching, respectively, of the long alkyl chains of stearic acid, are not present in the pure polypyrrole structure; they have been observed in the SPP structure. Moreover, a CH_2_ vibration band, emanating from the crystalline fatty acid structure, was observed within the SPP structure at approximately 720 cm^–1^.^22^ The thermal decomposition behavior of SPP5 particles was evaluated by thermogravimetric analysis (TGA) to confirm the presence of a polypyrrole coating. The most substantial weight loss, which amounted to approximately 56%, occurred within the temperature range of 225–260 °C. This loss was attributed to the thermal degradation of stearic acid.^49^ Subsequent weight loss between 310 and 352 °C was assigned to the degradation of the polypyrrole shell, which decomposes at a higher temperature^50^ (see Figure S1). The hydrophobicity of SPP5 was assessed through the measurement of the static contact angle (see Figure 1F). A water droplet deposited on the particle bed exhibited a contact angle in excess of 137.4 ± 1.1° (*n* = 40), indicating a hydrophobic surface. This outcome resulted from the combination of long-chain alkyl groups. In order to assess the light-to-heat conversion ability of the particles, SPP5 was irradiated with 808 nm near-infrared light (NIR) in cycles, and temperature changes were monitored during heating and cooling using a thermal camera (see Figure 1E). Reversible heating and cooling cycles confirmed the photothermal activity of the polypyrrole particles. The photothermal conversion efficiency was calculated as 33%.

### Synthesis and Characterization of Ferrofluid (FF)

Ferrofluid, a colloidal suspension of magnetic nanoparticles for use as a core liquid, has been synthesized. These structures offer significant advantages for stimulus-responsive LM due to their rapid response to magnetic fields. The ferrofluid synthesized in this study was stabilized with tetramethylammonium hydroxide. The hydrodynamic diameter of the particles was found to be 14.4 ± 4.8 nm by DLS (see Figure S4). The results demonstrated that the dispersion exhibited a monodisperse and stable distribution.^51^ The XRD patterns of the ferrofluid samples are displayed in Figure S5A. Subsequently, these patterns were compared to standard JCPDS diffraction patterns of magnetite (JCPDS 019-629). It is evident that all peak positions at (2 2 0), (3 1 1), (4 0 0), (5 1 1), and (4 4 0) are consistent with the standard data for the magnetite structure. Therefore, the XRD results provide evidence for the presence of magnetite-related crystal phases. Moreover, the EPR spectrum of ferrofluid was recorded (see Figure S5B) at ambient temperature to further investigate their magnetic characteristics, thereby demonstrating the typical superparamagnetic nature. The spectrum displays a predominant resonance signal with a *g* value of 2.14, which is in accordance with the results of previous studies on iron oxide-based nanoparticle systems.^52^ The broad line width (ΔHp ≈ 880 G) is indicative of strong dipole–dipole interactions and magnetic coupling between nanoparticles as previously reported in superparamagnetic systems.^53^ A weak resonance was observed at approximately 160 mT, corresponding to a *g* value of 4.3. This was associated with the glass capillary that was used during the measurement. The EPR results provide additional evidence that is consistent with the structural (XRD) analysis and support the magnetic nature of the ferrofluid system.

### Fabrication of Liquid Marbles with Modified Coating Particles

In this study, we investigate the potential of synthesized particles as liquid marble coating materials, in addition to their utilization in soft microrobotic applications. The ability of these particles to stabilize water droplets and form mobile, robust, and stimulus-responsive liquid marbles was evaluated. Using SPP and F-MNP and their combination (70% SPP and 30% F-MNP powder (CO)), we successfully prepared liquid marbles (CO LMs), and their stability was investigated (see Figures S6 and S7). It was observed that as the volume increased, the LM shape changed from a spherical form to an ellipsoidal form. This phenomenon can be attributed to the predominant effect of gravity on the surface tension in larger volumes.^54^ The stability of liquid marbles is determined by particle packing,^55^ hydrophobic properties,^56^ and interfacial attraction.^57^ The hydrophobic properties of SPP and F-MNP particles are significant factors in shell stability. The enhanced stability observed in F-MNP particles can be explained by the formation of a shell structure, primarily consisting of a jammed granular layer at the interface as a result of magnetic dipole interactions.^58^

In general, formed liquid marbles demonstrated a spherical geometry, stability, and mobility on solid substrates, demonstrating no leakage or deformation under ambient conditions. Digital photographs of liquid marbles are shown in Figure 2A–D. These results confirmed the particle’s capacity to function as a supportive shell in the liquid marble system.

**Figure 2:**
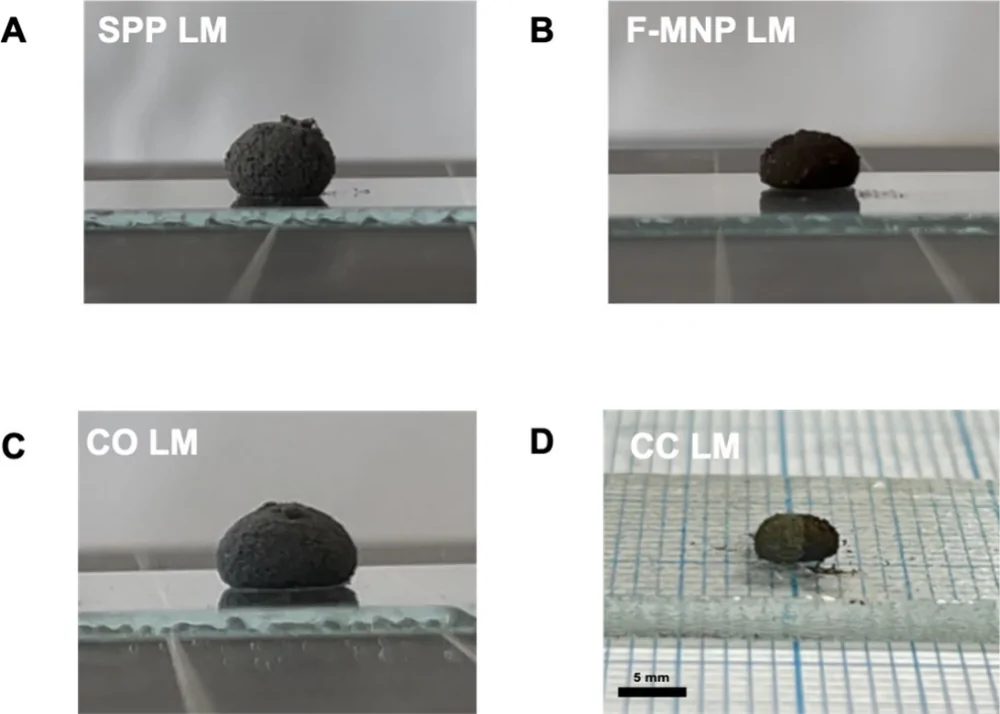
Digital photographs illustrating the different liquid marbles: (A) side view of the SPP LM, (B) side view of the F-MNP LM, (C) side view of the 70% SPP/30% F-MNP CO LM, and (D) side view of the compartmentalized liquid marble (CC LM).

To clarify the component contributions to liquid marble motion, representative control experiments were carried out (see Figure S8). The ferrofluid demonstrated a clear response to the applied magnetic field, confirming its intrinsic magnetic responsiveness as the core liquid. In contrast, a SPP-coated liquid marble lacking a magnetic component exhibited no observable locomotion when subjected to an external magnetic field. Additionally, a magnetic nanoparticle-coated liquid marble placed on a solid surface and exposed to near-infrared (NIR) irradiation showed no response. The photothermal behavior of the SPP LM was investigated through the application of NIR laser irradiation at a wavelength of 808 nm at a power of 1.5 W. As shown in Figure 3A following exposure to light, the LM exhibited a response to the stimuli, moving in a direction that was away from the source of the laser irradiation. The movement of photothermally responsive liquid marbles can be explained by the photothermal effect-induced Marangoni flow. This phenomenon is defined as the transfer of mass that occurs along an interface between two fluids in the presence of a gradient of surface tension. The SPP LM (Figure 3A) and CO LM (Figure 3C) have been demonstrated to absorb NIR light, thereby increasing the local temperature of the irradiated side and consequently generating a temperature gradient around the liquid marble. Under 808 nm NIR laser irradiation, the SPP LM and CO LM exhibited a localized temperature increase, as confirmed by infrared thermal imaging. For the SPP LM, the temperature difference that was measured was approximately 4.4 ± 0.2 °C, and for the CO LM, a slight increase in temperature difference of approximately 5.4 ± 0.3 °C was observed. Local heating at the irradiated side of the marble induced a surface tension gradient at the interface between the marble and the underlying water layer. The surface tension gradients for both LMs were calculated as 0.70 and 0.85 mN/m, respectively.^59^ These thermal gradients at the water surface, otherwise known as surface tension gradients, have the capacity to generate Marangoni flow.^60^ The generation of a thermocapillary flow (Marangoni flow) on the water surface has driven the marble away from the high-temperature region.^61,62^

**Figure 3:**
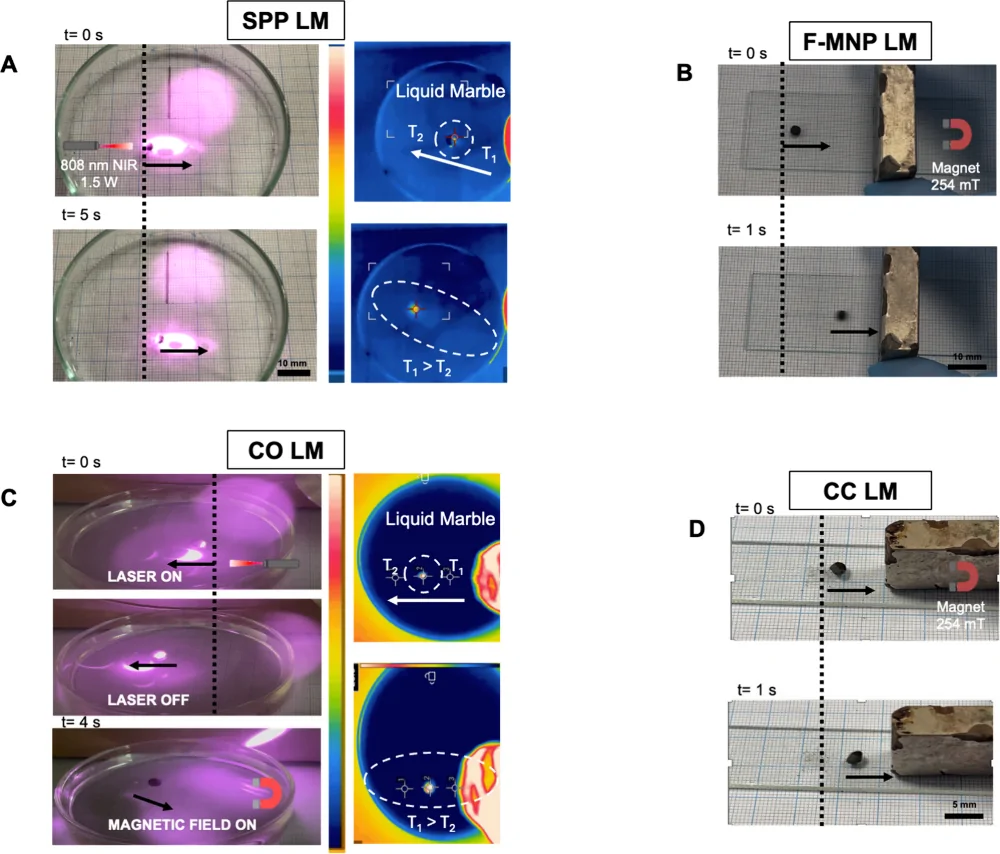
Representative optical and thermal images showing the motion of different liquid marble systems under external stimuli. (A) Top view optical and thermal camera images of the SPP LM motion on a planar water surface under a 1.5 W 808 nm NIR laser. (B) Top view optical images of the F-MNP LM sliding on a planar solid surface 254 mT magnetic field (on the magnet surface). (C) Top view optical and thermal camera images of the CO LM sliding on a planar water surface under dual stimuli (1.5 W 808 nm NIR laser and 254 mT magnetic field). (D) Top view optical images of CC LM locomotion on a planar solid surface under a 254 mT magnetic field (on the magnet surface).

In order to evaluate the magnetic actuation potential of the liquid marbles formed by using fluorinated magnetic nanoparticles (F-MNP LMs), motion tests were conducted on hydrophobic solid substrates under external magnetic fields at varying distances from the magnet (see Figure 4). The balance among magnetic driving forces, frictional resistance, and interfacial tension is a key factor governing liquid marble transport behavior.^63^

**Figure 4:**
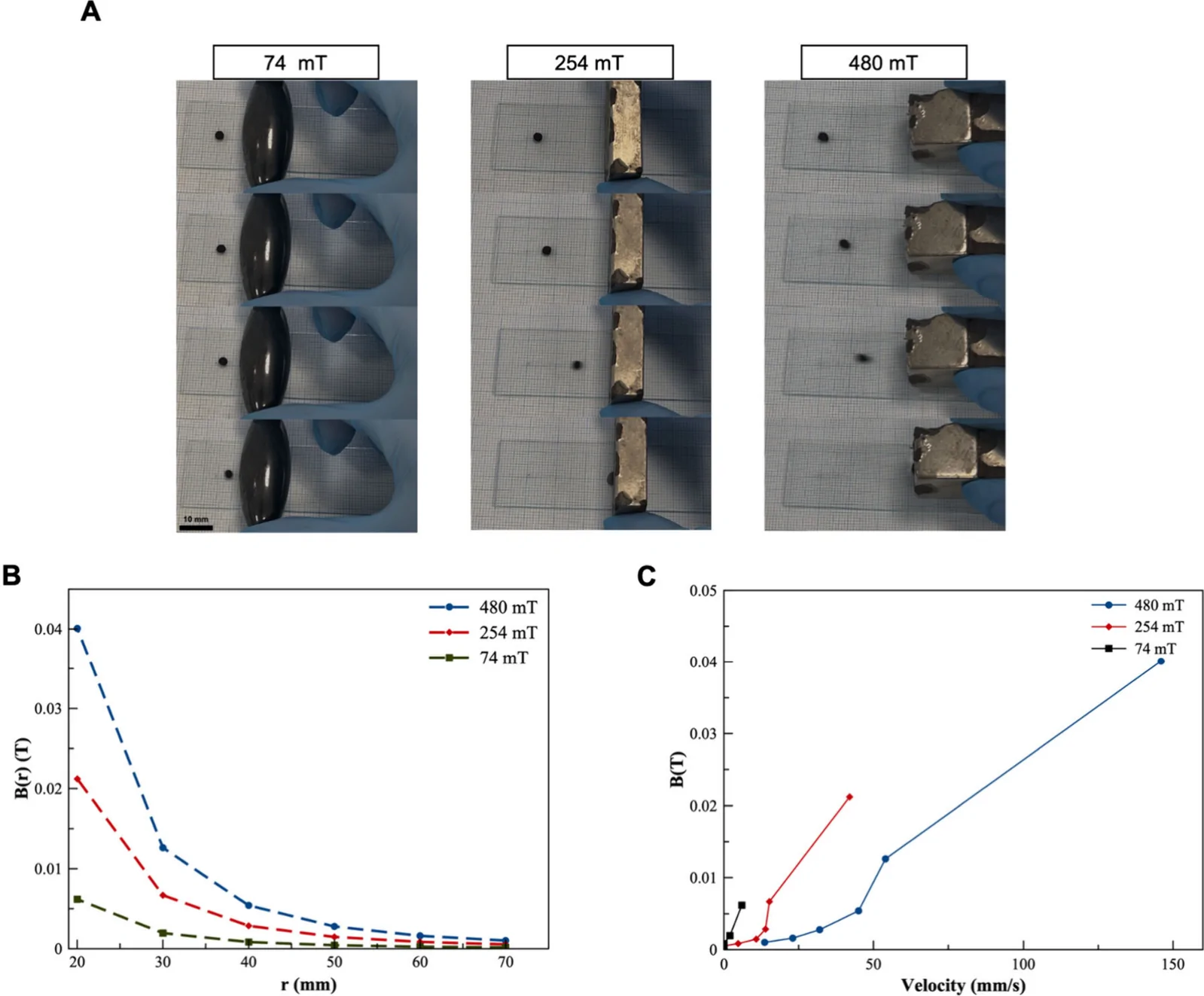
(A) Top view of F-MNP LM motion on a solid surface with different magnetic fields. (B) Magnetic field–distance profiles of the magnets used in the locomotion experiments. (C) Velocity of F-MNP LMs as a function of magnetic field strength.

Exposure of the marbles to a magnetic field using a magnet (∼254 mT) resulted in an instantaneous and directional movement, propelled by the magnetic body force exerted on the shell particles (Figure 3B). The coating exhibited high magnetization, which led to its rapid acceleration toward the magnetic source. To determine the practical operating range for liquid marble actuation, the relationship between magnetic flux density and distance was examined. As shown in Figure 4B, all magnets exhibited a similar trend dependent on distance, with a particularly significant decrease in magnetic field intensity within the range of 20–40 mm. Consequently, the velocity of the liquid marbles decreased as the magnetic field strength decreased (see Figure 4C). It is noteworthy that the 480 mT magnet produced the highest measured field, which resulted in the highest recorded velocity of approximately 150 mm/s. Higher magnetic field strengths were found to generate stronger magnetic driving forces acting on the liquid marbles that were responsive to the magnetic field. The correlation observed between the measured magnetic field applied to the liquid marble and the resultant velocity provides quantitative support for the role of magnetic actuation in the locomotion mechanism. Despite the high actuation efficiency at a higher magnetic field strength, precise motion control was challenging. It has been observed that the magnetic shell demonstrated a swift reaction to the applied external force, frequently resulting in the marble being abruptly pulled toward the magnet. This resulted in a phenomenon of partial disintegration or structural collapse of the marble structures, particularly in the regions exhibiting high fields or when the magnet was positioned close to the structure. This phenomenon is attributed to the nonuniform force distribution across the shell and the lack of internal cohesion between the solid shell and the liquid core under rapid motion.^64^ The construction of a compartmentalized architecture (CC LM) involved the mixing of two liquid marbles with two different functional coating materials. This process resulted in the formation of shell domains that were physically segregated (Figure 2D). As shown in Figure 3D, the CC LM exhibited a selective magnetic actuation and light responsiveness on solid substrates. Conversely, it demonstrated rapid destabilization and dispersion in liquid environments. The interfacial energies on the liquid marble are subject to change when it is positioned on a water surface. In systems where the coating layer contains particles that are weakly bound, there is a possibility of water penetration and liquid marble desorption.^65^ The behavior of CC LM on a water surface is presumably due to the asymmetric distribution of magnetic particles and the resultant imbalance in interfacial forces. In order to address the absence and thereby create a dual-stimulus-responsive liquid marble to integrate the benefits of both photothermal and magnetic actuation mechanisms on different surfaces, a composite liquid marble (CO LM) was formulated (see Figure 2C). The exposure of a composite marble to an 808 nm near-infrared (NIR) laser resulted in directional movement on the water surface, following the thermal gradient generated by localized photothermal heating. Simultaneously, the magnetic responsiveness induced by F-MNPs facilitated magnetically guided movement. It is noteworthy that incorporating NIR-responsive SPP particles into the F-MNP-based liquid marble shell introduced both light responsiveness and enhanced mechanical stability to the marbles. The presence of particles of varying sizes and surface properties within a liquid marble has been suggested to enhance shell packing and interfacial jamming. This stabilization facilitated the observation of magnetically actuated motion on water surfaces, which proved to be a successful outcome. The composite formulation thus expands the operational environment of magnetic liquid marbles from solid substrates to floating aqueous systems. This may provide a more extensive range of applications in soft robotics and lab-on-liquid platforms for liquid marbles. The utilization of a combination of light and magnetic fields in a sequential manner has enabled the demonstration of the multimodal controllability of the composite marbles. It has been demonstrated that movement driven by light provides fine directional control, rendering it ideal for slow or precise manipulation. In contrast, magnetic actuation has been proposed as a method for enabling rapid repositioning or retrieval over longer distances (see Figure 3C).

The velocities of SPP liquid marbles exhibited a correlation with the laser beam diameter and power (Figure 5A). In the presence of a constant laser intensity, the generation of smaller spot sizes (0.2–1.0 cm) resulted in an increase in velocity. On the other hand, as the beam diameter increased, velocity decreased due to a dilution of the thermal gradient. It was determined that the optimal speed value for SPP LM was 0.68 ± 0.2 mm/s, with a laser power of 1.5 W and a beam diameter of 1.0 cm in a 50 μL liquid marble. F-MNP liquid marbles on a solid surface were manipulated with magnets of three different strengths (74, 254, and 480 mT). As presented in Figure 5B, the experiment yielded findings that indicated a proportional increase in the velocity of the liquid marbles with an increase in the magnetic field strength.^23^ It is challenging to regulate the movement of liquid marbles at increased magnetic field strengths, despite their high velocity advantage, because of the proximity of coating particles to the magnet and the tendency of magnetic nanoparticles to be attracted to strong magnetic fields.

**Figure 5:**
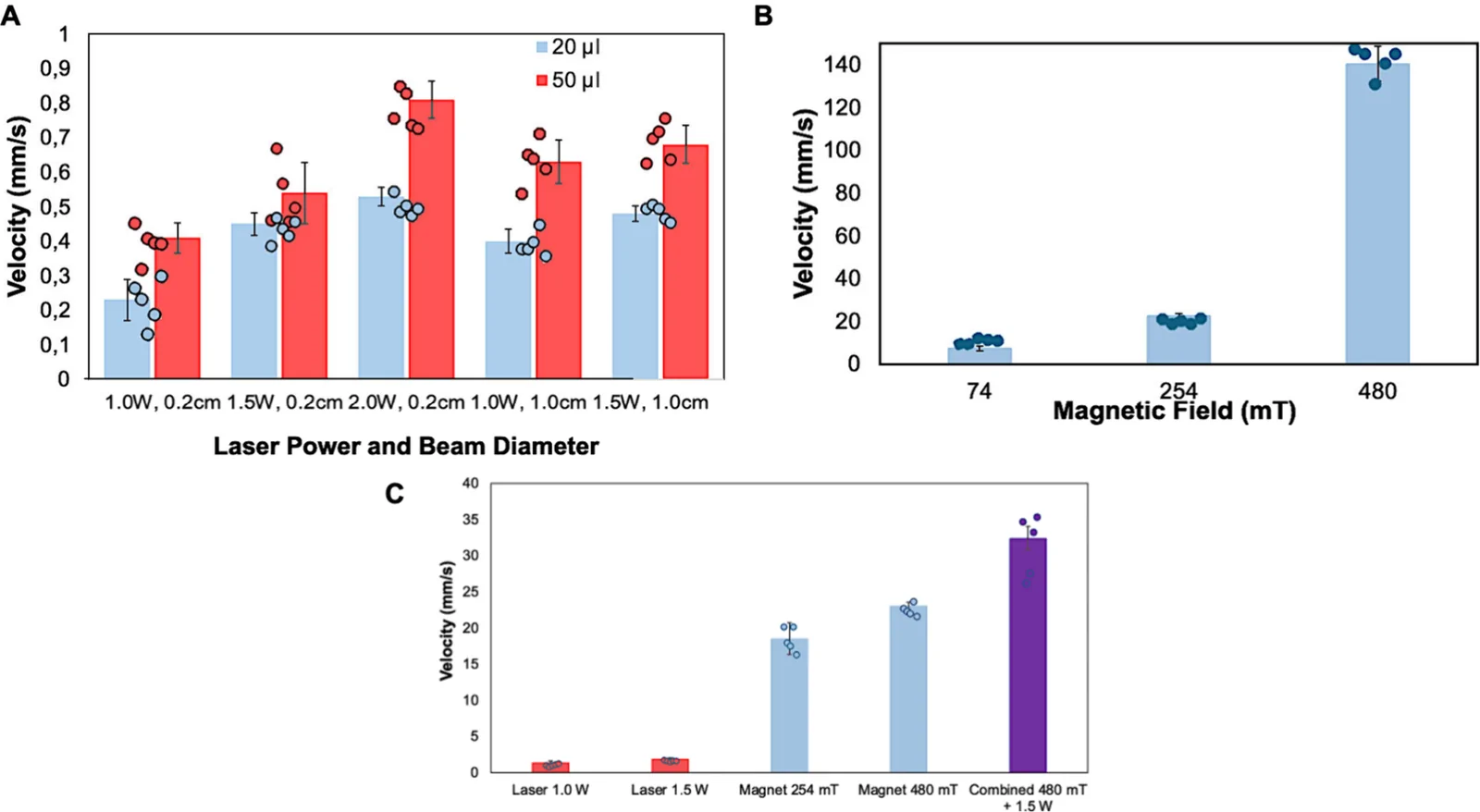
Velocity analysis of liquid marbles under different actuation mechanisms. (A) Velocity of the SPP LM under different laser powers and beam diameters. (B) Effect of magnetic field strength on the F-MNP LM velocity. (C) Velocity of the CO LM under different laser powers, magnetic field strengths, and dual stimuli. Data are presented as the mean ± standard deviation derived from five independent measurements (*n* = 5).

Subsequently, initial investigations focused on the movement of individual liquid marbles, utilizing stimuli to understand each stimulus’s distinct actuation mechanism on the CO LM on the water surface. NIR light was first applied to the LM. The application of NIR light resulted in the observation of a movement of the CO LM, facilitated by the SPP present in its structure. Furthermore, it was observed that, in contrast to the F-MNP LM, the CO LM was stable on the water surface and exhibited a tendency to move in the direction of the magnetic field when a magnet was applied. As expected, due to the reduced number of magnetic nanoparticles in the structure compared to F-MNP LM, the speed of CO LM was found to be lower but more controllable. As is evident from the results, the prepared CO LM can be stimulated separately under both a magnetic field and NIR light. In addition, the response capabilities of the system to dual stimuli were observed by applying both stimuli to a single CO LM and observing the combined motion of 30 ± 4.2 mm/s (see Figure 5C). The interplay of these two driving mechanisms results in an enhanced net propulsion force, with magnetic forces acting as a guiding and pulling force on the marble, while photothermal effects generate interfacial stress gradients, thereby reducing resistance and promoting interfacial flow. Conclusively, it was determined that the functional CO LMs demonstrated sensitivity to both NIR light and magnetic fields.

Evidently, the facilitation of external stimulus responsiveness by coating engineering has been shown to result in a reliance on the shell alone, thereby restricting the system’s internal dynamics. To broaden the boundaries of controllability and responsiveness, the subsequent research concentrated on a system in which both the outer shell and the liquid core function actively. Utilizing ferrofluids to create the dual-function liquid marbles has resulted in an innovative system capable of responding to multiple stimuli via independent mechanisms. The ferrofluid droplets were coated using SPP particle powders, and liquid marbles (SPP FLMs) were obtained. The liquid marbles obtained in the study were observed to remain stable for 1 h (see Figure S9).

The photothermal SPP shell has been shown to absorb near-infrared (808 nm) laser irradiation and convert it into localized heat. This results in a temperature gradient at the liquid–liquid interface between the marble and the water surface (see Figure 6D), with an average temperature difference of approximately 9.0 °C between the irradiated and nonirradiated regions. It is calculated that this temperature difference results in an estimated surface tension difference of approximately 1.44 mN/m. This induced Marangoni flow propels the marble away from the laser source as shown in Figure 6B. Simultaneously, the ferrofluidic core, composed of superparamagnetic nanoparticles suspended in a carrier liquid, responds to external magnetic fields via magnetophoretic attraction, thereby enabling directional controlled motion toward a magnet as shown in Figure 6A. In opposition to earlier liquid marble systems, in which either the shell or the core predominated as the actuation mechanism, this configuration offers complementary and collaborative actuation pathways. When both stimuli were applied either sequentially or simultaneously, enhanced control over direction, velocity, and trajectory was achieved (see Figure 6E). As demonstrated in Figure 6C, the SPP FLM structure exhibits a speed that is 10 times higher than that of the CO LM structures. Furthermore, we observed that the FLM speed that has been achieved is greater than that reported in existing ferrofluid liquid marble studies.^64^ This phenomenon can be attributed to the SPP FLM being more affected by the magnetic field due to the ferrofluid core liquid. This phenomenon is also observed in the context of laser actuation, resulting in the observed acceleration of particles.

**Figure 6:**
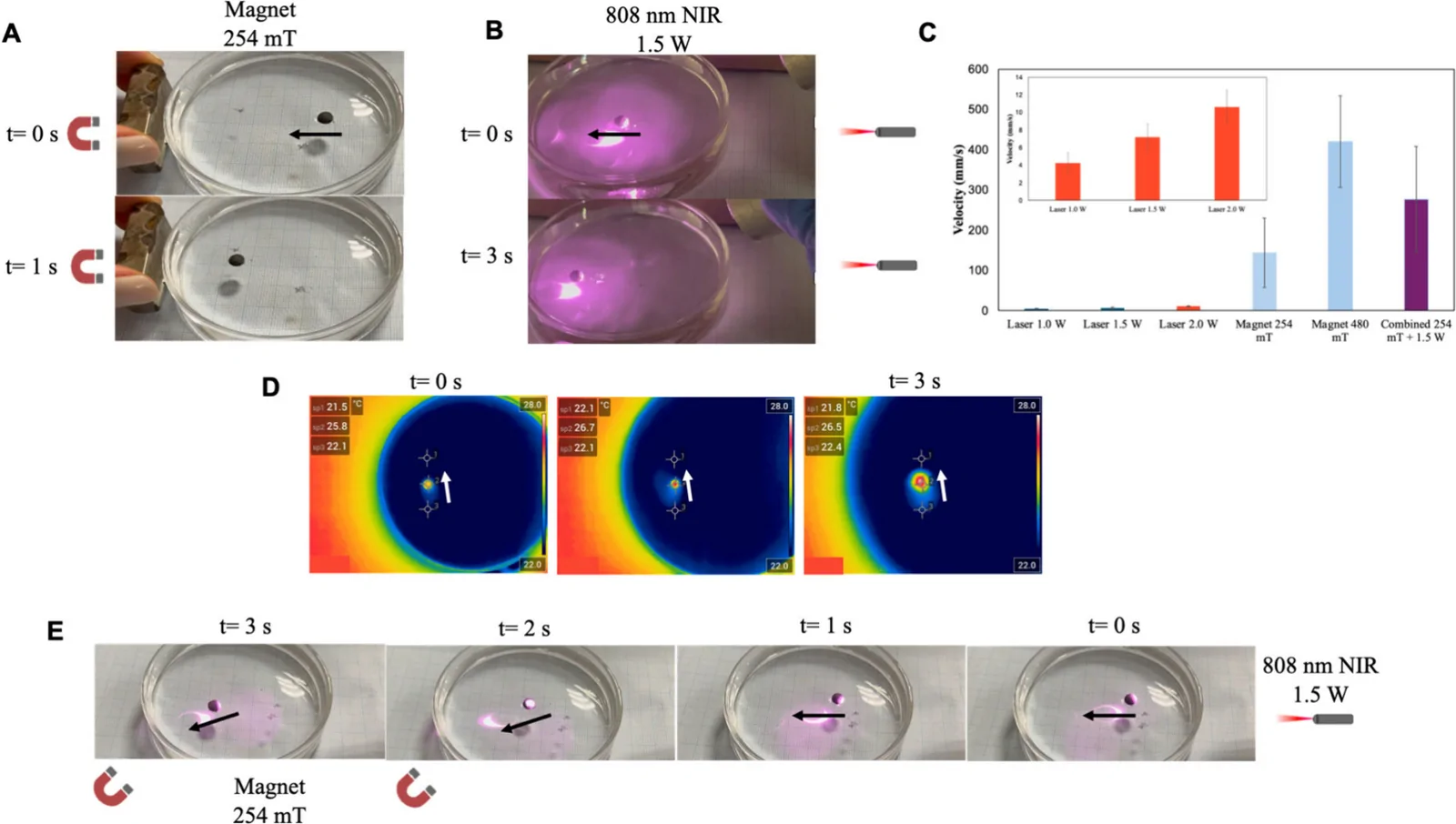
(A) Top view of the the SPP FLM sliding on a planar water surface under a 254 mT magnetic field. (B) Top view of the SPP FLM sliding on a planar water surface under a 1.5 W 808 nm NIR laser. (C) Velocity of the SPP FLM under different actuation mechanisms. (D) Thermal camera images of SPP FLM motion on a planar water surface under a 1.5 W 808 nm NIR laser. (E) Top view of the SPP FLM sliding on a planar water surface under dual stimuli (1.5 W 808 nm NIR laser 254 mT magnetic field).

The integrated responsiveness of the internal and external elements not only extends the operational capacity of these marbles but also highlights the potential of this system as a proof-of-concept platform for the manipulation of liquid marbles under multiple external stimuli.

### Application of Stimulus-Responsive Liquid Marbles as Soft Microrobots

A micromotor should demonstrate the capacity for locomotion and controllability, as well as the capability to perform work at the microscale.^66^ Liquid marbles are capable of acting as mobile microscale carriers due to their particle-stabilized interface, low friction, and ability to respond to external stimuli. This enables them to be remotely guided across surfaces. The present system can demonstrate programmable and dual-stimulus-responsive motion at liquid–soft interfaces. The recent literature has indicated that such systems are widely recognized as soft microrobots due to their controllable, adaptive, and task-oriented locomotion behaviors.^67,68^ In order to evaluate the performance of the designed liquid marbles under various environmental conditions, their directional movement within specially designed channels was further investigated. These channels, constructed on layers of pure water and polyacrylamide (PAM) hydrogel, serve as simplified models of soft environments, mimicking tissue-like or microfluidic architectures. Two types of composite marbles, CO LM and SPP FLM, were subjected to external stimuli, including near-infrared (NIR) light and magnetic fields. The movement of the marbles along these defined pathways demonstrates their capacity to function as autonomous, stimulus-responsive soft microrobots, thereby exhibiting the capability of navigating over complex or soft interfaces.

As illustrated in Figure 7, the directional locomotion of composite liquid marbles composed of 70% SPP and 30% F-MNP particles on polyacrylamide (PAM) hydrogel-based microchannels is presented. In the single-stimulus mode, the marble is directed along a predetermined linear trajectory by either photothermal or magnetic actuation, operating individually. Nevertheless, in the dual-stimulus mode, whereby both a laser and a magnet are applied concurrently, the marble displays enhanced velocity and maneuverability, thereby demonstrating the cooperative effect of dual-stimulus actuation (see Movie S1). It was observed that the motion behavior of liquid marbles on the PAM surface was noticeably slower than that observed on the water surface (see Figure 7B). This can be explained by interfacial and mechanical characteristics of the hydrogel environment. The low resistance exhibited by water surfaces allows for enhanced mobility of liquid marbles in the presence of interfacial tension imbalances caused by photothermal heating or magnetic force imbalances. In contrast, the PAM hydrogel provides a more constrained and mechanically resistive interface, which can reduce LM motion. However, despite the decrease in velocity, the directional locomotion of the marbles was clearly observed. The polyacrylamide was selected for this study with the intention of demonstrating programmable and controllable LM navigation within a confined soft material environment. The aim of using the hydrogel-based platform was to provide a more application-relevant platform for investigating guided transport, directional control, and localized chemical interactions under various conditions. This observation indicates that even in the presence of significant mechanical limitations, the marbles exhibited adequate responsiveness to external stimuli.

**Figure 7:**
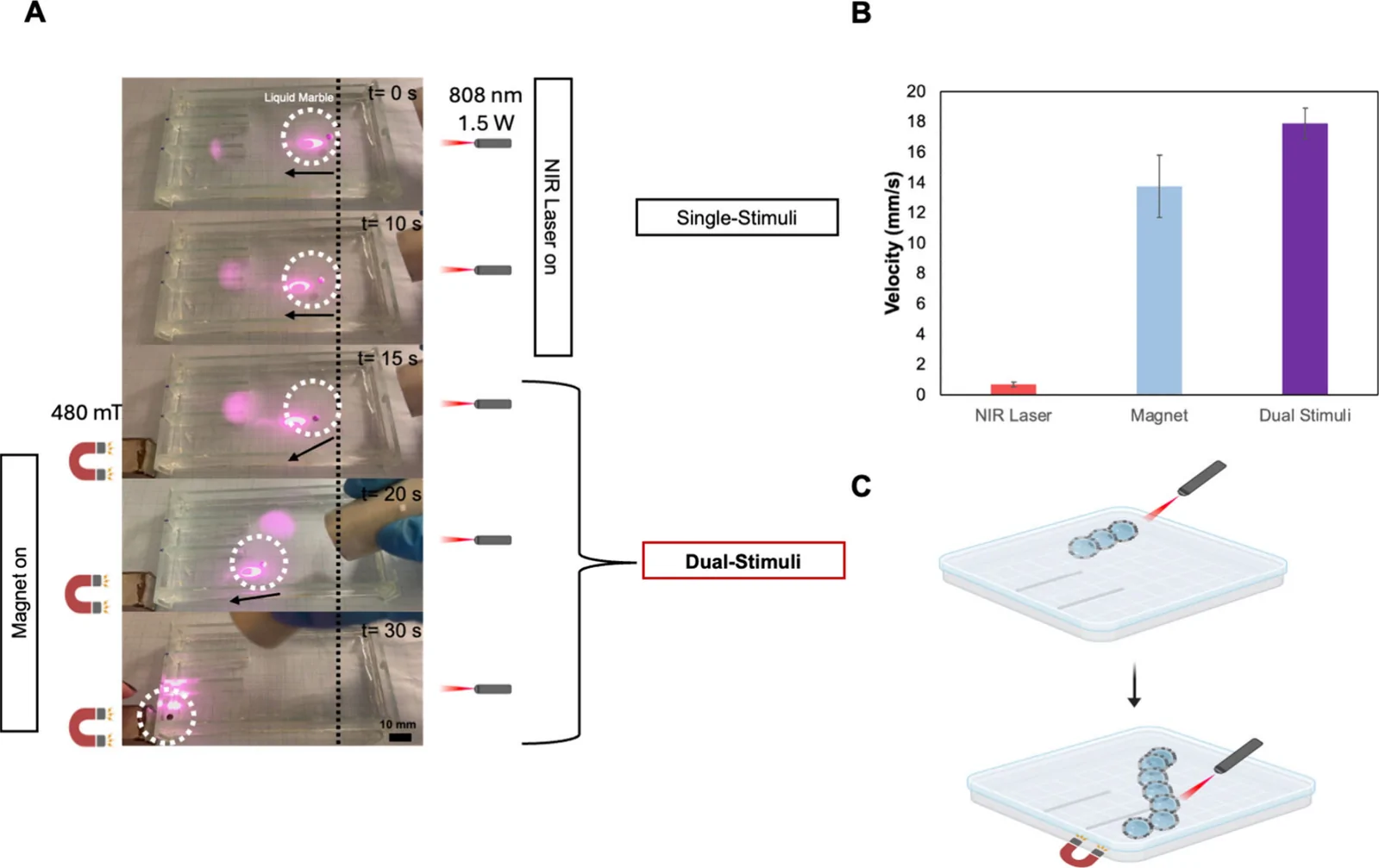
(A) Trajectory of guided motion of the CO LM on a polyacrylamide surface with NIR irradiation, a magnetic field, and combined dual stimuli. (B) Velocity of the CO LM on a PAM surface under different actuation mechanisms. (C) Schematic representation of the movement of the CO LM on a PAM surface under external stimulation.

The objective of this study was to demonstrate the full functional potential of liquid marbles as untethered microrobotic platforms. The SPP FLM configuration consists of a ferrofluid-based inner phase, offering magnetic responsiveness, encapsulated within a shell composed of photothermally active SPPs. The presence of two orthogonal actuation pathways allows for precise control and programming of motion across PAM hydrogel surfaces (see Figure 8A and Movie S2). Two permanent magnets were used to demonstrate the effect of the magnetic field strength on the motion behavior of liquid marbles. As the two magnets had different magnetic field strengths (74 and 480 mT) and distributions, the liquid marbles exhibited different motion responses. As observed in the Co LM, the locomotion velocity of the SPP FLM on the PAM substrate was lower than that observed on a planar water surface (see Figure 8B). However, the SPP FLM exhibited a slightly higher velocity on the PAM surface compared with that of the Co LM. This behavior is related to the magnetic responsiveness of the ferrofluid core, enabling an effective external manipulation and directional guidance under magnetic stimulation. The magnetic field may contribute to the reduction of interfacial resistance within the hydrogel environment, thereby enhancing the controllability of the movement. It is particularly noteworthy that exposure to extended NIR laser radiation results in the degradation or softening of the SPP particle shell, ultimately leading to the disruption of the marble’s integrity. This structural failure results in the controlled release of the internal cargo, thereby demonstrating the feasibility of on-demand delivery. This release mechanism can be calibrated by modulating the laser intensity and exposure duration, enabling the delivery to be spatially and temporally targeted in microreactors or biosensing applications. In addition to actuation, these marbles display a pH-sensitive optical behavior. The color transition that was observed was determined by the interaction between the ferrofluid core that had been released by the ruptured liquid marble and the phenolphthalein indicator that was present on the PAM substrate (see Figure S10). The color change in phenolphthalein, from colorless to pink, has been observed to be dependent on the pH of the environment. Under acidic and neutral conditions, it remains colorless, but under alkaline conditions, it turns a pink color. As a result, the localized alkalinity change or local chemical environment change consistent with the phenolphthalein response after LM rupture induces a visible color change in the surrounding region. This results in the marble functioning not only as a mobile unit but also as a proof of concept of an environmentally responsive optical sensor, capable of detecting chemical changes in its surroundings.

**Figure 8:**
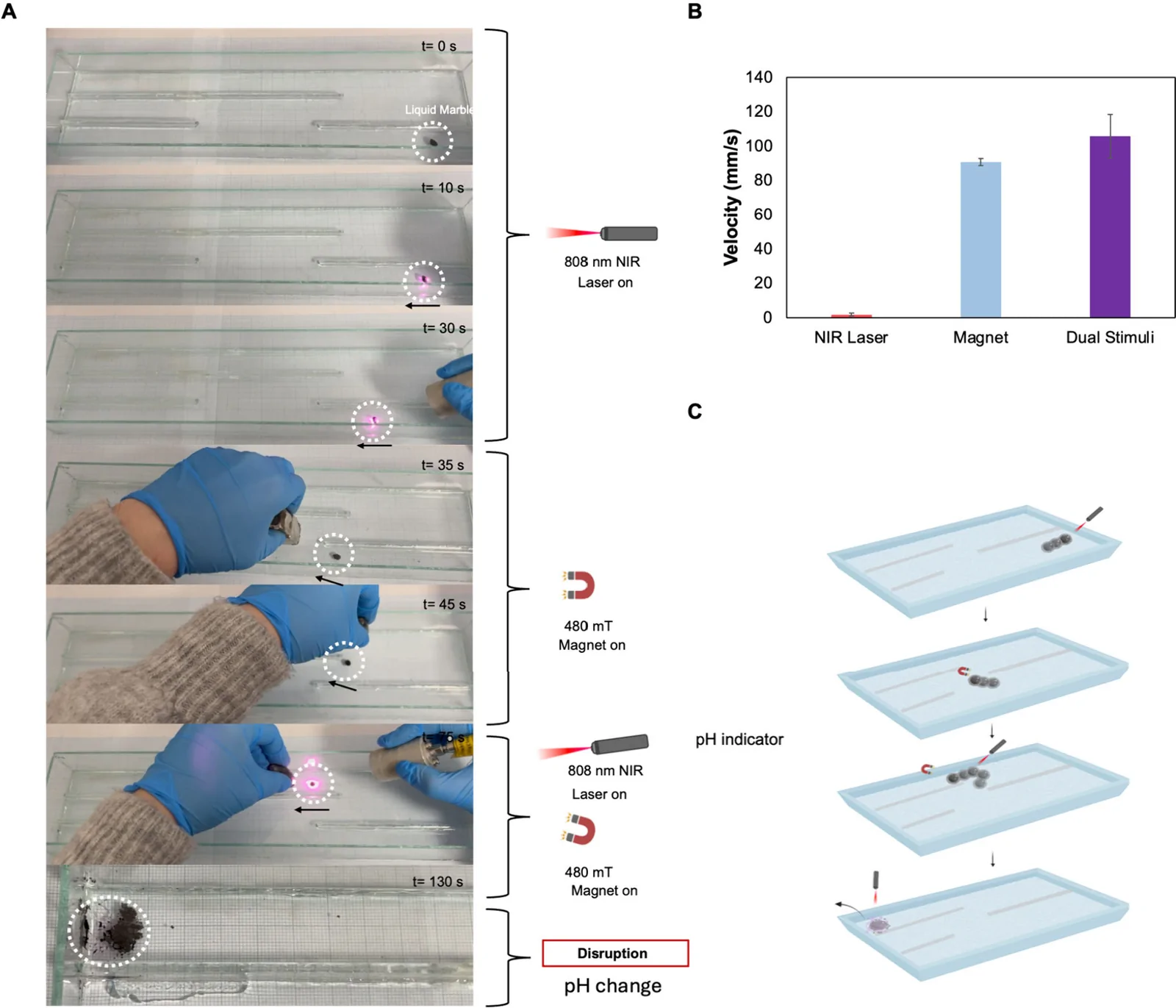
(A) Trajectory of guided motion and the pH-triggered optical response of ferrofluid liquid marbles on a polyacrylamide surface under NIR irradiation, a magnetic field, and combined dual stimuli. (B) Velocity of the SPP FLM on a PAM surface under different actuation mechanisms. (C) Schematic representation of the movement of the SPP FLM on a PAM surface under external stimulation.

Collectively, these results highlight the multifunctional potential of the dual-stimulus liquid marble system. The system provides an experimental basis as a soft microrobot, which possesses the capability to facilitate programmable locomotion, chemical sensing, and external stimulus-triggered release. Consequently, this provides new possibilities for lab-on-a-drop systems, intelligent microcarriers, and soft robotic actuators that function on water or soft substrates.

## Conclusions

In the present study, the development and investigation of dual-stimulus-responsive liquid marbles have been systematically studied for microrobotic applications. By integration of two functional particle types, photothermally active SPP particles and hydrophobic magnetic nanoparticles (F-MNPs), with a ferrofluid core, hybrid LMs that can respond to light and magnetic fields independently and synergistically were created. The coupling of the nanoparticle shell and the ferrofluidic interior has been demonstrated to enable the observation of controlled Marangoni-driven motion under NIR irradiation and precise magnetic guidance. This robust locomotive behavior was systematically investigated on water and polyacrylamide (PAM) hydrogel surfaces. The results demonstrated that the surrounding interface has a strong influence on the locomotion behavior. Overall, this work expands the capabilities of LMs from passive droplet encapsulation to actively controlled micromotors. The proposed dual-stimulus integration provides a foundation for future soft robotic architecture, chemical or therapeutic delivery platforms, and intelligent microfluidic components.

## Supplementary Material







## Data Availability

The data that support the findings of this study are available from the corresponding author upon reasonable request.

## Notes

Thermogravimetric analysis of SPP; FTIR spectra of SPP and stearic acid; VSM analysis of F-MNP; DLS analysis of synthesized ferrofluid nanoparticles; XRD and EPR analysis of ferrofluid; stability analysis of SPP, F-MNP, and SPP FLM liquid marbles with digital camera images; control experiments for actuation; and investigation of pH-induced color change on PAM surfaces with digital camera images (PDF)
Dual-stimulus-responsive motion of the CO LM under both magnetic field and NIR irradiation on a PAM-filled channel (video shown at 5× playback speed) (Movie S1) (MP4)
Dual-stimulus-responsive motion of the SPP FLM under two different magnetic fields (74 and 480 mT) and NIR irradiation on a PAM-filled channel (video shown at 7× playback speed) (Movie S2) (MP4)

## References

[1] Sreejith K. R., Gorgannezhad L., Jin J., Ooi C. H., Stratton H., Dao D. V., Nguyen N.-T. (2019). Liquid marbles as biochemical reactors
for the polymerase chain reaction. Lab Chip..

[2] Moragues T., Arguijo D., Beneyton T., Modavi C., Simutis K., Abate A. R., Baret J.-C., deMello A. J., Densmore D., Griffiths A. D. (2023). Droplet-based
microfluidics. Nat. Rev. Methods Primers.

[3] Nguyen N.-K., Singha P., Dai Y., Rajan Sreejith K., Tran D. T., Phan H.-P., Nguyen N.-T., Ooi C. H. (2022). Controllable
high-performance liquid marble micromixer. Lab
Chip..

[4] Nguyen N.-K., Ooi C. H., Singha P., Jin J., Sreejith K. R., Phan H.-P., Nguyen N.-T. (2020). Liquid Marbles as Miniature Reactors
for Chemical and Biological Applications. Processes.

[5] Aussillous P., Quéré D. (2001). Liquid marbles. Nature..

[6] Aussillous P., Quéré D. (2006). Properties
of liquid marbles. Proc. R. Soc. A.

[7] Alp G., Alp E., Aydogan N. (2020). Magnetic liquid marbles to facilitate rapid manipulation
of the oil phase: Synergistic effect of semifluorinated ligand and
catanionic surfactant mixtures. Colloids Surf.
A: Physicochem. Eng. Asp..

[8] Dayyani H., Mohseni A., Bijarchi M. A. (2024). Dynamic
behavior of floating magnetic
liquid marbles under steady and pulse-width-modulated magnetic fields. Lab Chip..

[9] Kavokine N., Anyfantakis M., Morel M., Rudiuk S., Bickel T., Baigl D. (2016). Light-Driven
Transport of a Liquid Marble with and against Surface
Flows. Angew. Chem., Int. Ed..

[10] Asaumi Y., Rey M., Vogel N., Nakamura Y., Fujii S. (2020). Particle Monolayer-Stabilized
Light-Sensitive Liquid Marbles from Polypyrrole-Coated Microparticles. Langmuir.

[11] Wang S., Si W., Zhao Z., Zhou W., He Y., Guo Z. (2025). Superamphiphobic
light-driven liquid marbles. Chem. Eng. J..

[12] Akbari M. J., Edrisnia H., Sarkhosh M. H., Bijarchi M. A., Shafii M. B. (2025). Modeling
the post-impact dynamics of liquid marbles on a hydrophilic surface:
Investigating bounces and oscillation. Phys.
Rev. Fluids..

[13] Ooi C. H., Van Nguyen A., Evans G. M., Gendelman O., Bormashenko E., Nguyen N.-T. (2015). A floating self-propelling liquid
marble containing aqueous ethanol solutions. RSC Adv..

[14] Wang Q., Xiang N., Lang J., Wang B., Jin D., Zhang L. (2024). Reconfigurable Liquid-Bodied Miniature Machines: Magnetic Control
and Microrobotic Applications. Adv. Intell.
Syst..

[15] Yang W., Wang X., Wang Z., Yuan Z., Ge Z., Yu H. (2023). A multi-stimulus-responsive bionic fish microrobot for remote intelligent
control applications. Soft Matter.

[16] Okmen
Altas B., Goktas C., Topcu G., Aydogan N. (2024). Multi-Stimuli-Responsive
Tadpole-like Polymer/Lipid Janus Microrobots for Advanced Smart Material
Applications. ACS Appl. Mater. Interfaces..

[17] Wu R., Zhu Y., Cai X., Wu S., Xu L., Yu T. (2022). Recent Process
in Microrobots: From Propulsion to Swarming for Biomedical Applications. Micromachines.

[18] Toprak B., Kalaycioglu G. D., Aydogan N. (2025). Multifunctional CD-MOF Hybrid Systems:
Integrating Drug Delivery, Photothermal Therapy, and Nanozyme Applications. Small.

[19] Ye M., Zhou Y., Zhao H., Wang Z., Nelson B. J., Wang X. (2023). A Review of Soft Microrobots: Material, Fabrication, and Actuation. Adv. Intell. Syst..

[20] Sun H. C. M., Liao P., Wei T., Zhang L., Sun D. (2020). Magnetically
Powered Biodegradable Microswimmers. Micromachines.

[21] Sultan U., Kotulla C., Kefle K., Fujii S., Vogel N. (2025). Rough and
Tough: How Particle Surface Roughness Affects Liquid Marble Formation
and Stability. Adv. Sci..

[22] Tsumura Y., Oyama K., Fameau A.-L., Seike M., Ohtaka A., Hirai T., Nakamura Y., Fujii S. (2022). Photo/Thermo Dual Stimulus-Responsive
Liquid Marbles Stabilized with Polypyrrole-Coated Stearic Acid Particles. ACS Appl. Mater. Interfaces..

[23] Gu Y., Shoukat T., Jin Y., Yang J., Jiang L., Ashalley E., Wang C., Reyes D., Yu P., Wang Z. (2025). Light-Driven
Rotational Control of Ferrofluid Liquid
Marbles in Magneto-Optical Traps for Optical and Acoustic Modulation. Adv. Opt. Mater..

[24] Park J., Kim J.-y., Pané S., Nelson B. J., Choi H. (2021). Acoustically
Mediated Controlled Drug Release and Targeted Therapy with Degradable
3D Porous Magnetic Microrobots. Adv. Healthc.
Mater..

[25] Kunanopparatn A., Hayashi M., Atsuta Y., Iwata Y., Yamamoto K., Matsui K., Hirai T., Nakamura Y., Unob F., Imyim A. (2024). pH-Responsive Liquid Marbles Stabilized with Chitosan-Stearic
Acid-Conjugated Particles. ACS Sustain. Chem.
Eng..

[26] Wang J., Zheng J., Zhang C., Duan Y., Zhang Y., Guo H., Qiu Y., Wang X., Liu L., Yu H. (2026). 4D-Printed
Microrobots with Synergistic Integration of Magnetic Actuation, Diffractive
Optical Sensing, and pH-Adaptive Morphing. Adv.
Funct. Mater..

[27] Bielas R., Kubiak T., Molcan M., Dobosz B., Rajnak M., Józefczak A. (2024). Biocompatible Hydrogel-Based Liquid Marbles with Magnetosomes. Materials.

[28] Oehlsen O., Cervantes-Ramírez S. I., Cervantes-Avilés P., Medina-Velo I. A. (2022). Approaches on Ferrofluid Synthesis and Applications:
Current Status and Future Perspectives. ACS
Omega.

[29] Mohammadrashidi M., Azizian P., Bijarchi M. A., Shafii M. B. (2023). Vibration
and Jumping
of Ferrofluid Marbles under an Initial Magnetic Perturbation. Langmuir.

[30] Yang J., He Y., Jiao F., Wang M. (2022). Reciprocating Oscillation of a Floating
Ferrofluid Marble Triggered by Magnetic Fields. Langmuir.

[31] Bormashenko E., Pogreb R., Bormashenko Y., Musin A., Stein T. (2008). New Investigations
on Ferrofluidics: Ferrofluidic Marbles and Magnetic-Field-Driven Drops
on Superhydrophobic Surfaces. Langmuir.

[32] Sarkhosh M.
H., Yousefi M., Dabirzadeh M. H., Bijarchi M. A., Nejat
Pishkenari H. (2025). Experimental investigation of reciprocating ferrofluid
marble motion driven by repulsive and attractive magnetic forces:
A scheme for mixing enhancement during transportation. J. Mol. Liq..

[33] Bielas R., Kubiak T., Kopčanský P., Šafařík I., Józefczak A. (2023). Tunable particle
shells of thermo-responsive liquid
marbles under alternating magnetic field. J.
Mol. Liq..

[34] Sarkhosh M. H., Edrisnia H., Bijarchi M. A., Pishkenari H. N. (2026). Magnetic
manipulation of non-magnetic liquid marbles via ferrofluid marbles
using switchable attractive and repulsive forces: Dynamics and on-demand
position control. J. Mol. Liq..

[35] Tenjimbayashi M., Mouterde T., Roy P. K., Uto K. (2023). Liquid marbles: review
of recent progress in physical properties, formation techniques, and
lab-in-a-marble applications in microreactors and biosensors. Nanoscale.

[36] Kawashima H., Paven M., Mayama H., Butt H.-J., Nakamura Y., Fujii S. (2017). Transfer of Materials
from Water to Solid Surfaces Using Liquid Marbles. ACS Appl. Mater. Interfaces..

[37] Bormashenko E., Bormashenko Y., Oleg G. (2010). On the Nature of the Friction between
Nonstick Droplets and Solid Substrates. Langmuir.

[38] Barman N., Jain K., Borbora A., Kumar S., Tenjimbayashi M., Manna U. (2025). Regulating the Self-Propulsion
of Liquid Marbles on a Water Pool. Adv. Funct.
Mater..

[39] Zhao Y., Xu Z., Parhizkar M., Fang J., Wang X., Lin T. (2012). Magnetic liquid
marbles, their manipulation and application in optical probing. Microfluid. Nanofluid..

[40] Tang S.-Y., Sivan V., Khoshmanesh K., O’Mullane A. P., Tang X., Gol B., Eshtiaghi N., Lieder F., Petersen P., Mitchell A. (2013). Electrochemically
induced actuation of liquid metal marbles. Nanoscale.

[41] Fujii S., Yusa S.-i., Nakamura Y. (2016). Stimuli-Responsive
Liquid Marbles:
Controlling Structure, Shape, Stability, and Motion. Adv. Funct. Mater..

[42] Tenjimbayashi M., Samitsu S., Watanabe Y., Nakamura Y., Naito M. (2021). Liquid Marble
Patchwork on Super-Repellent Surface. Adv. Funct.
Mater..

[43] Alp E., Aydogan N. (2016). A comparative study: Synthesis of superparamagnetic
iron oxide nanoparticles in air and N2 atm. Colloids Surf. A: Physicochem. Eng. Asp..

[44] Berger P., Adelman N. B., Beckman K. J., Campbell D. J., Ellis A. B., Lisensky G. C. (1999). Preparation and
Properties of an Aqueous Ferrofluid. J. Chem.
Educ..

[45] Kappes M., Friedrich B., Pfister F., Huber C., Friedrich R. P., Stein R., Braun C., Band J., Schreiber E., Alexiou C. (2022). Superparamagnetic Iron Oxide Nanoparticles for Targeted
Cell Seeding: Magnetic Patterning and Magnetic 3D Cell Culture. Adv. Funct. Mater..

[46] Zhang L., He R., Gu H.-C. (2006). Oleic acid
coating on the monodisperse magnetite nanoparticles. Appl. Surf. Sci..

[47] Huang C., Jiao F., He Y., Yang J., Zhao H. (2025). On the motion
of a liquid marble sliding on the liquid-infused surface. J. Mol. Liq..

[48] Martins C., Rolo C., Cacho V. R. G., Pereira L. C. J., Borges J. P., Silva J. C., Vieira T., Soares P. I. P. (2025). Enhancing
the
magnetic properties of superparamagnetic iron oxide nanoparticles
using hydrothermal treatment for magnetic hyperthermia application. Mater. Adv..

[49] Kar T., Samanta A., Sinnur H., Kumar M. (2022). Studies on Effect of
Application of Capric Acid and Stearic Acid based Reactive Phase Change
Materials (rPCM) with PHAMS Binder on Thermal Comfort of Cotton Khadi
Fabric as Thermo-tropic Smart Textiles. J. Nat.
Fibers.

[50] Ashraf S. M., Ahmad S., Riaz U. (2004). Pseudothermoset
blends of poly (methyl
methacrylate) and polypyrrole morphological, thermal, and conductivity
studies. J. Appl. Polym. Sci..

[51] Meng X., Qiu X., Zhao J., Lin Y., Liu X., Li D., Li J., He Z. (2019). Synthesis
of ferrofluids using a chemically induced
transition method and their characterization. Colloid Polym. Sci..

[52] Shahane G., Zipare K., Pant R. (2013). Synthesis
and characterization of
superparamagnetic Fe3O4 nanoparticles for ferrofluid application. Magnetohydrodynamics.

[53] Kubiak T., Dobosz B. (2025). Road Map for the Use of Electron Spin Resonance Spectroscopy
in the Study of Functionalized Magnetic Nanoparticles. In Materials.

[54] Jiang W., Guan X., Liu W., Li Y., Jiang H., Ngai T. (2025). Rotating Liquid Marble Microreactors
for Enhanced Enzymatic Reactions. Adv. Funct.
Mater..

[55] Saczek J., Murphy K., Zivkovic V., Putranto A., Pramana S. S. (2024). Impact
of coating particles on liquid marble lifetime: reactor engineering
approach to evaporation. Soft Matter.

[56] Zang D., Chen Z., Zhang Y., Lin K., Geng X., Binks B. P. (2013). Effect of particle hydrophobicity
on the properties
of liquid water marbles. Soft Matter.

[57] Singha P., Nguyen N.-K., Sreejith K. R., An H., Nguyen N.-T., Ooi C. H. (2021). Effect of Core Liquid Surface Tension
on the Liquid
Marble Shell. Adv. Mater. Interfaces..

[58] Wu X., Streubel R., Liu X., Kim P. Y., Chai Y., Hu Q., Wang D., Fischer P., Russell T. P. (2021). Ferromagnetic liquid
droplets with adjustable magnetic properties. Proc. Natl. Acad. Sci. U. S. A..

[59] Vargaftik N. B., Volkov B. N., Voljak L. D. (1983). International
Tables of the Surface
Tension of Water. J. Phys. Chem. Ref. Data.

[60] Uda M., Kawashima H., Mayama H., Hirai T., Nakamura Y., Fujii S. (2021). Locomotion
of a Nonaqueous Liquid Marble Induced by Near-Infrared-Light
Irradiation. Langmuir.

[61] Kawashima H., Mayama H., Nakamura Y., Fujii S. (2017). Hydrophobic polypyrroles
synthesized by aqueous chemical oxidative polymerization and their
use as light-responsive liquid marble stabilizers. Polym. Chem..

[62] Xu L., Ma C., Guan B., Lin J., Xiong K., Mou F., Luo M., Guan J. (2020). NIR light-steered
magnetic liquid marbles with switchable
positive/negative phototaxis. Appl. Mater. Today..

[63] Lehmann U., Hadjidj S., Parashar V. K., Vandevyver C., Rida A., Gijs M. A. M. (2006). Two-dimensional
magnetic manipulation
of microdroplets on a chip as a platform for bioanalytical applications. Sens. Actuators B: Chem..

[64] Mohammadrashidi M., Bijarchi M. A., Shafii M. B., Taghipoor M. (2023). Experimental
and Theoretical Investigation on the Dynamic Response of Ferrofluid
Liquid Marbles to Steady and Pulsating Magnetic Fields. Langmuir.

[65] Ravi A. S., Dalvi S. V. (2022). Unraveling stability
of a floating liquid marble, its
opening and resulting collapse patterns. Colloids
Surf. A: Physicochem. Eng. Asp..

[66] Zhou Y., Ye M., Hu C., Qian H., Nelson B. J., Wang X. (2023). Stimuli-Responsive
Functional Micro-/Nanorobots: A Review. ACS
Nano.

[67] Wang X., Lin D., Zhou Y., Jiao N., Tung S., Liu L. (2022). Multistimuli-Responsive
Hydroplaning Superhydrophobic Microrobots with Programmable Motion
and Multifunctional Applications. ACS Nano.

[68] Jeon H., Park K., Sun J.-Y., Kim H.-Y. (2025). Particle-armored
liquid robots. Sci. Adv..

