# Supplementary material for: Dual-Stimulus-Responsive Liquid Marbles with Programmable Motion on Liquid and Hydrogel Surfaces
Source: Langmuir. 2026 Jul 24;42(33):24364–76. doi: 10.1021/acs.langmuir.6c03198 (PMC13523737; doi:10.1021/acs.langmuir.6c03198)
Supplement: Supplementary file 1 [file la6c03198_si_001.pdf]

Supporting Information

Dual-Stimulus Responsive Liquid Marbles with Programmable Motion on Liquid and Hydrogel Surfaces

*Cansu Goktas[1], Nihal Aydogan[1]**

[1]Department of Chemical Engineering, Hacettepe University, Beytepe 06800 Ankara, Turkey

*Corresponding Author: Nihal Aydogan

Fax: +90 312 2992124,

Phone: +90 312 2976781

E-mail:anihal@hacettepe.edu.tr

Movie S1. Dual stimuli responsive motion of the CO LM under both magnetic field and NIR irradiation on a PAM filled channel (video shown at 5× playback speed)

Movie S2. Dual stimuli responsive motion of the SPP FLM under both two different magnetic field (74 and 480 mT) and NIR irradiation on PAM filled channel (video shown at 7× playback speed)

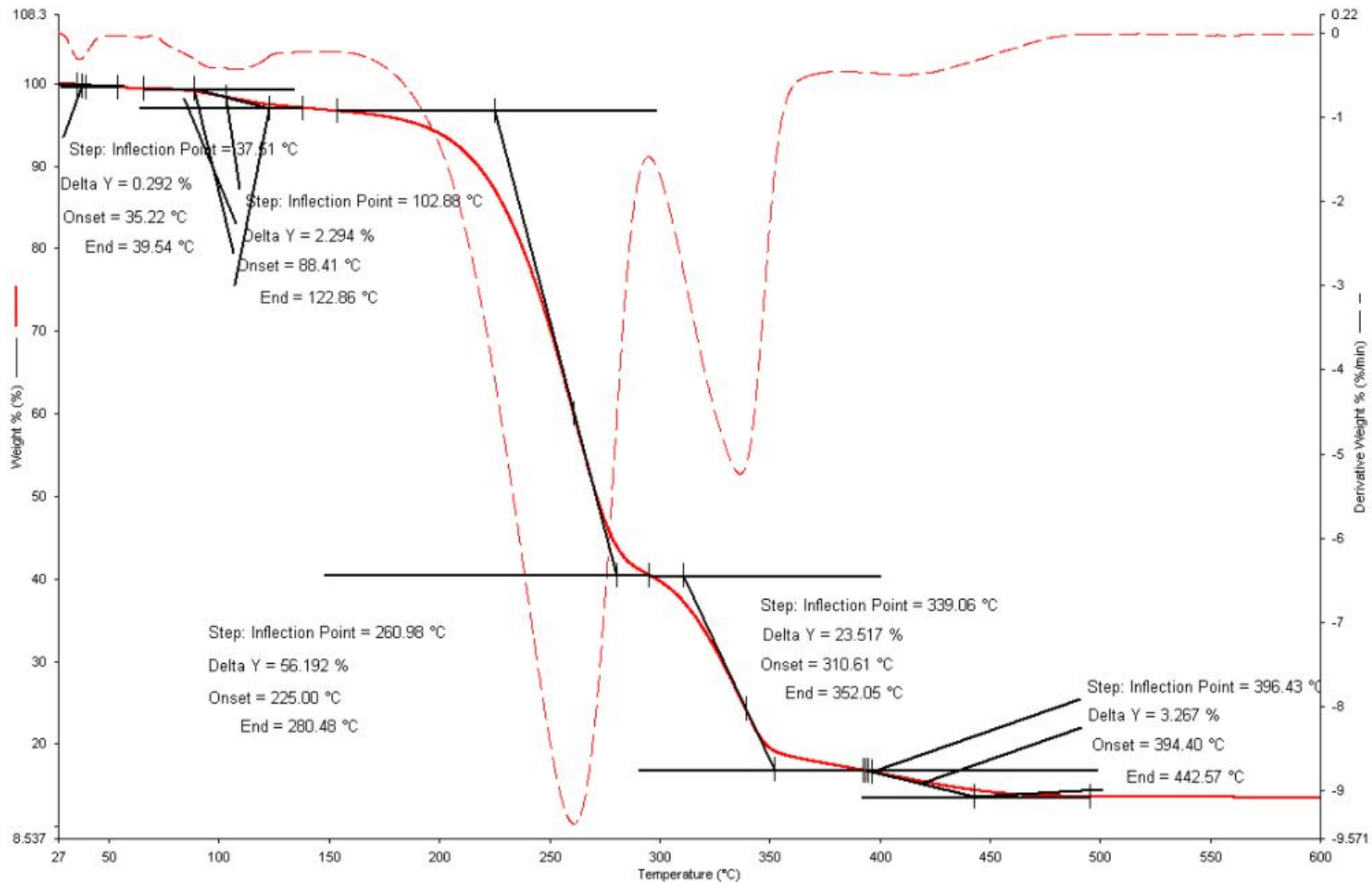

Figure S1. Thermogravimetric analysis (TGA) of SPP

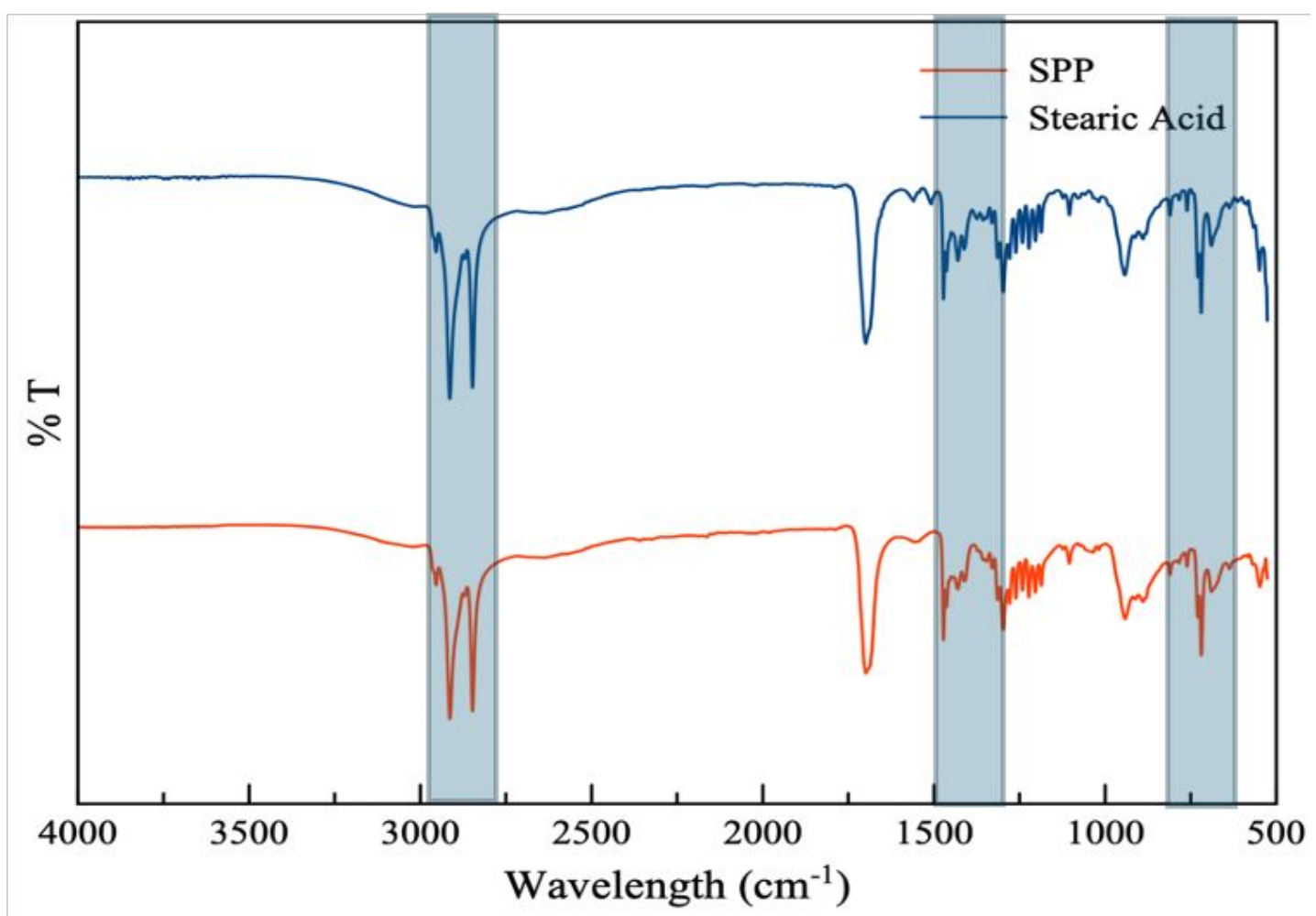

Figure S2. Fourier transform infrared (FTIR) spectra of SPP and stearic acid

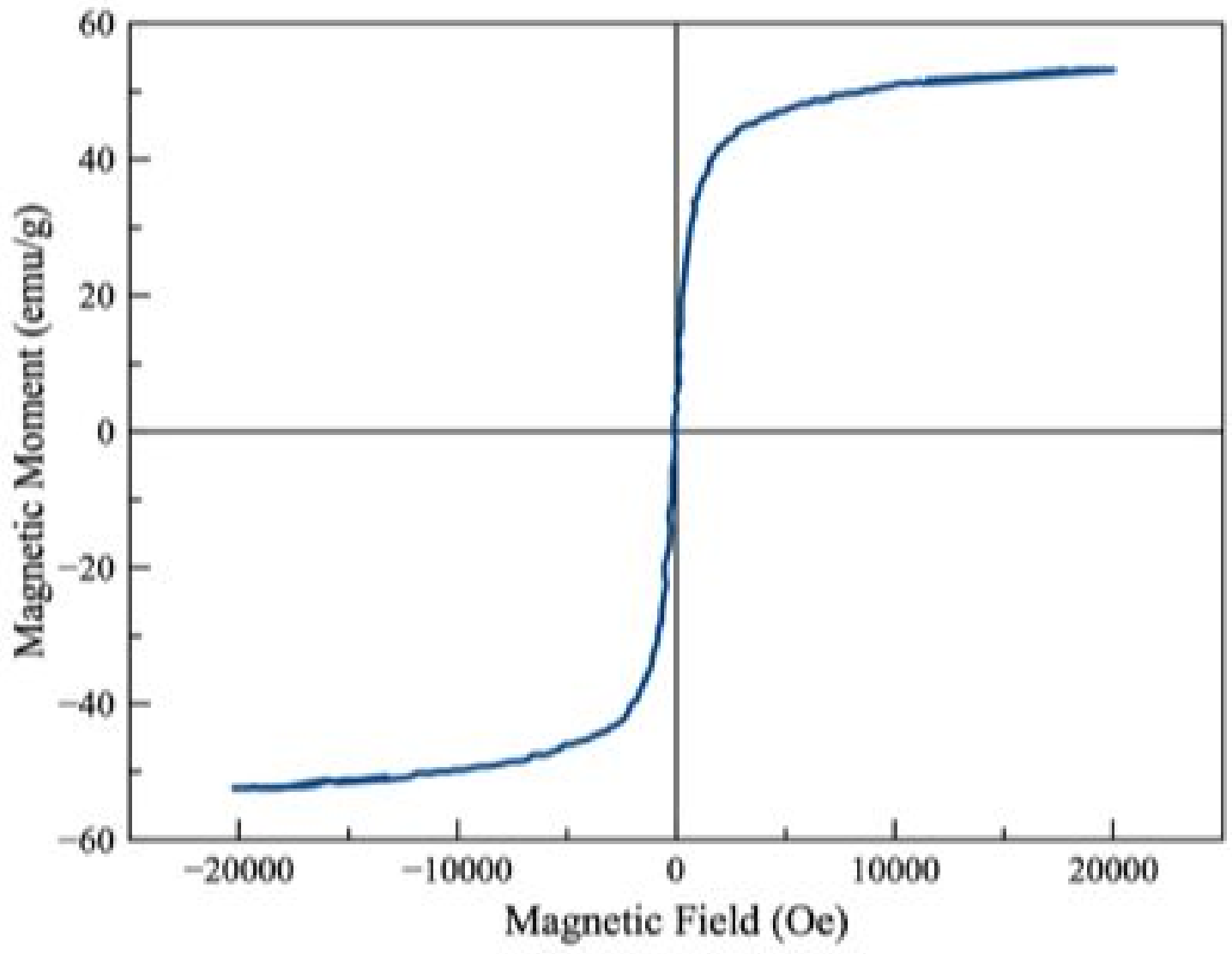

Figure S3. Magnetization curve of F-MNPs at room temperature

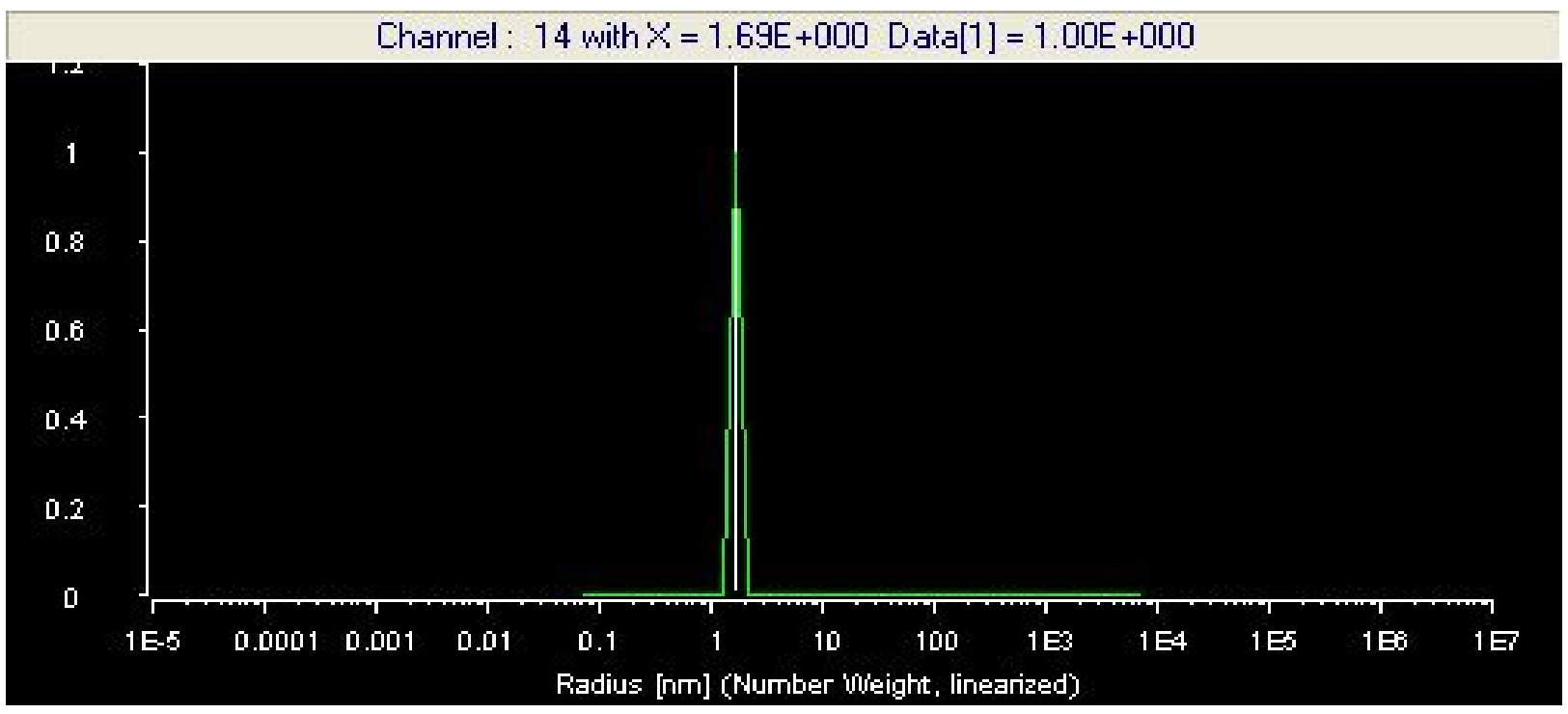

Figure S4. The hydrodynamic radius of nanoparticles in Ferrofluid

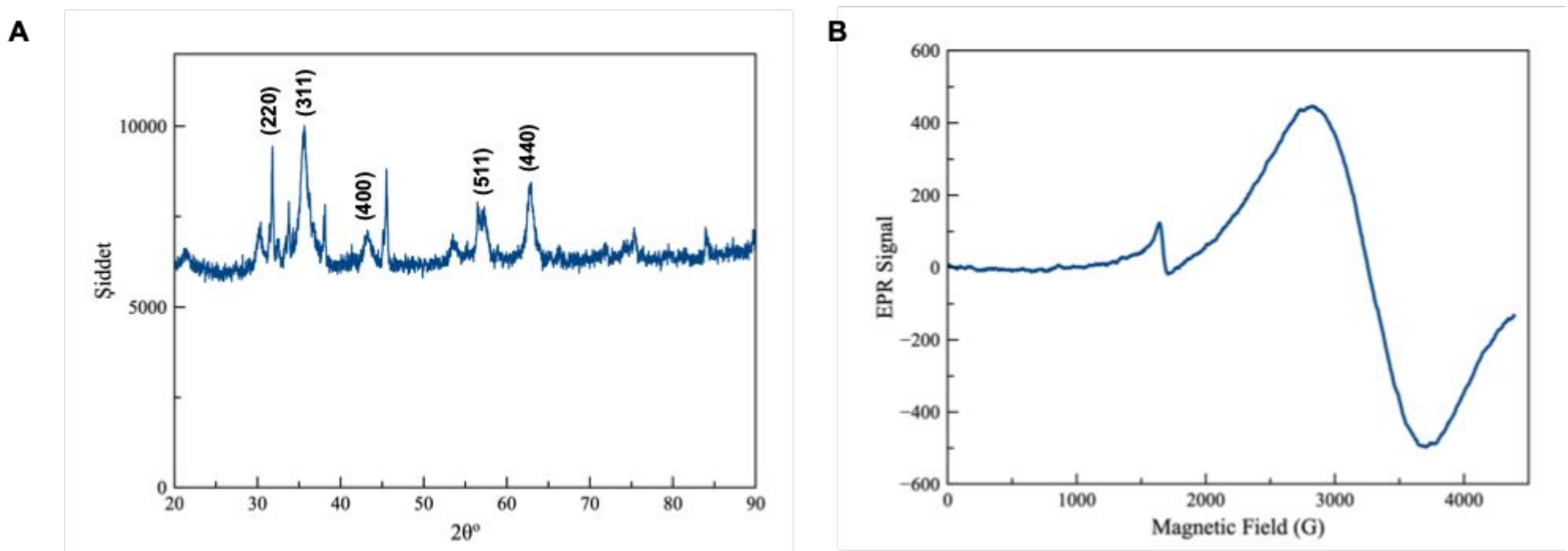

Figure S5. A) XRD pattern of ferrofluid B) EPR spectra of ferrofluid

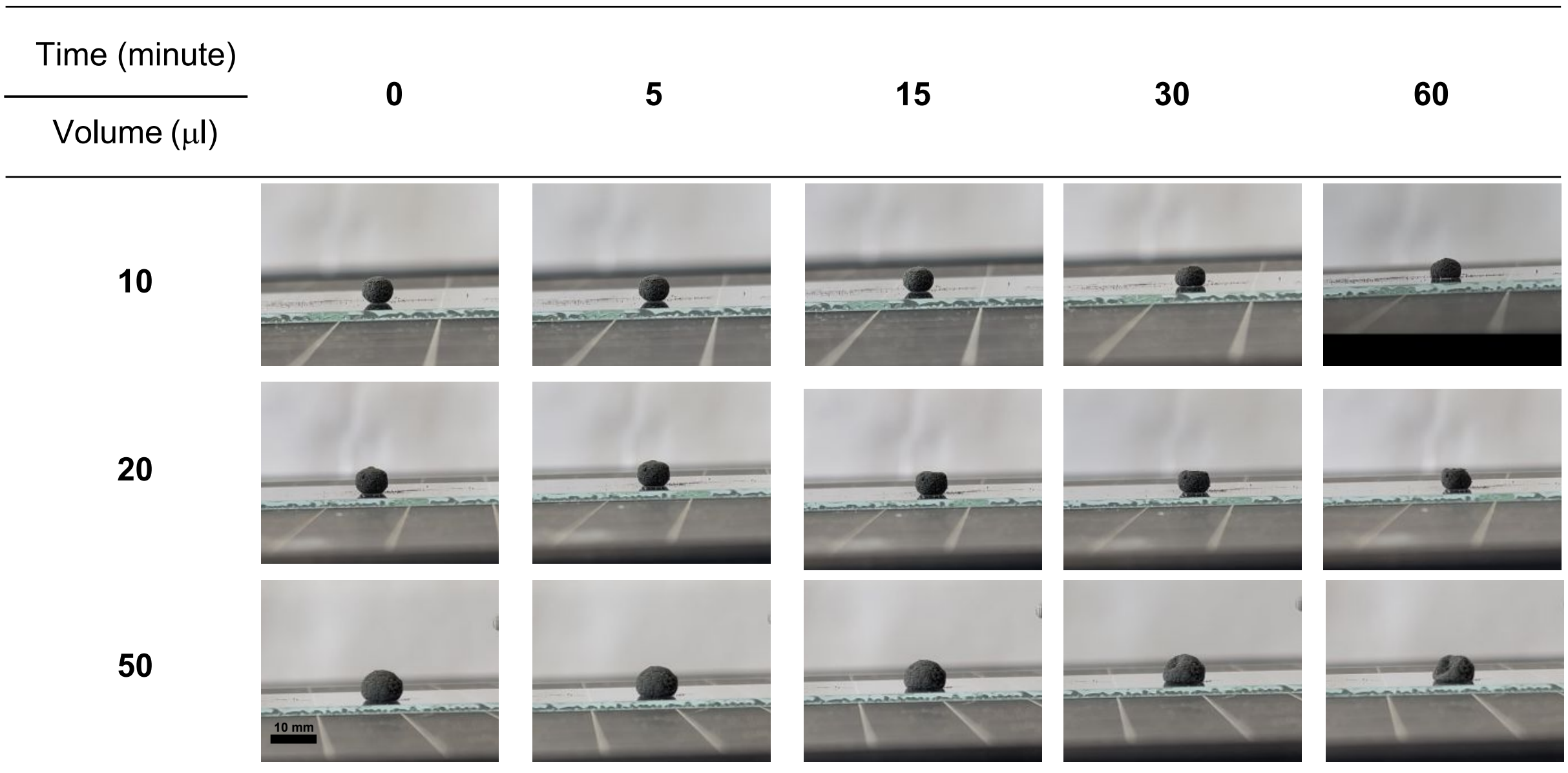

Figure S6. Investigation the stability of SPP liquid marble in three different volumes

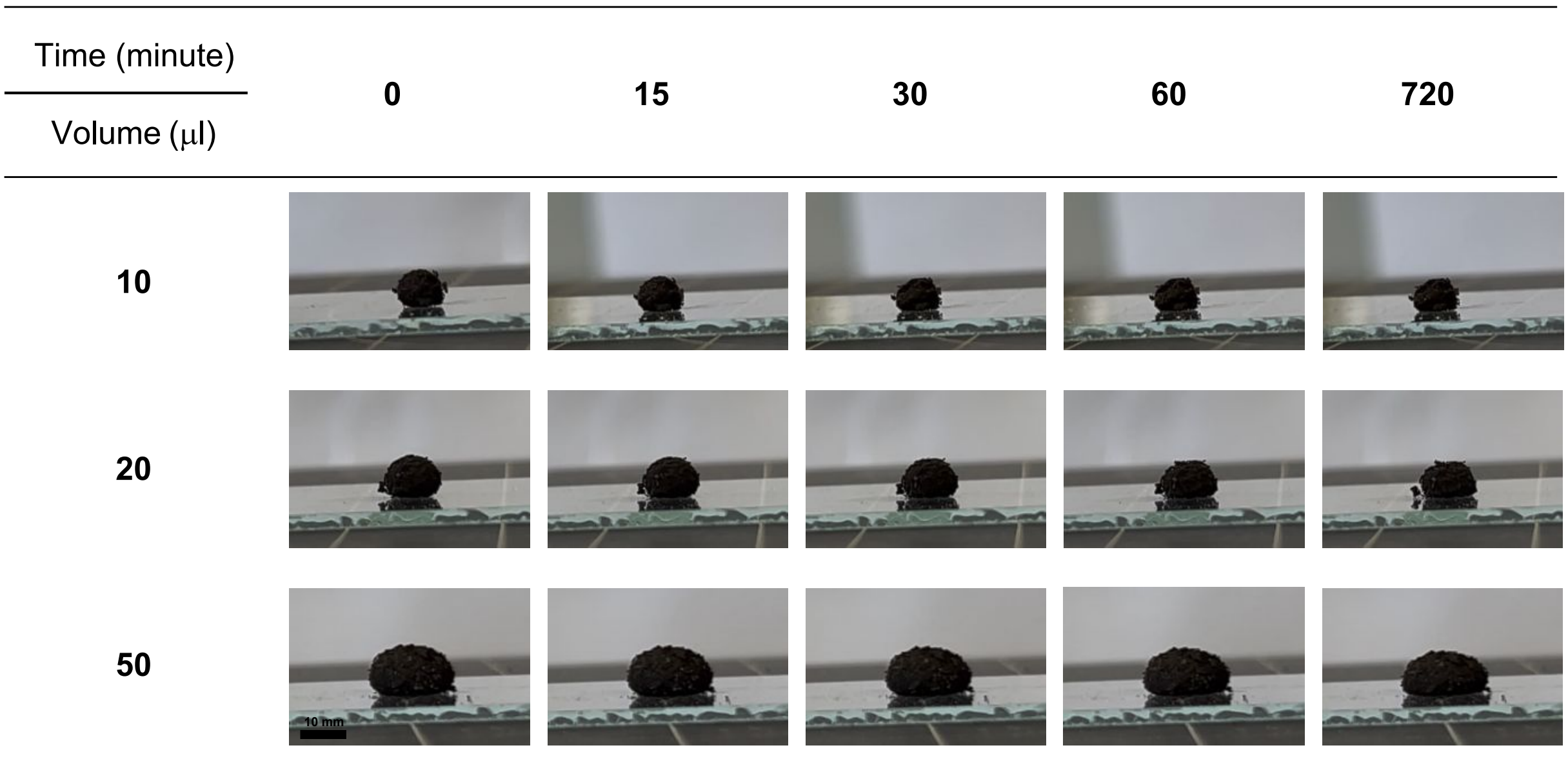

Figure S7. Investigation the stability of F-MNP liquid marble in three different volumes

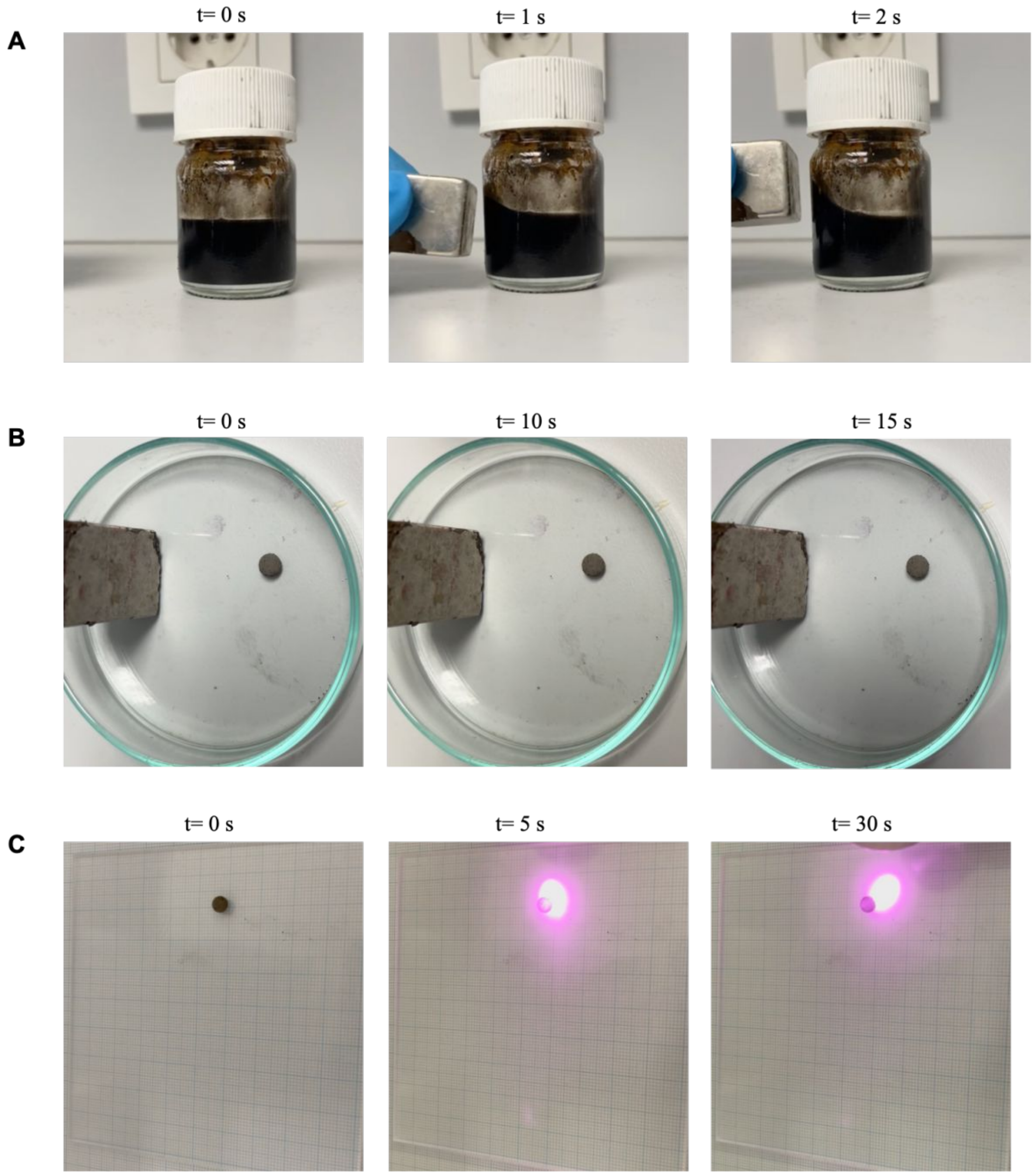

Figure S8. Control experiments for actuation of components A) Ferrofluid response to magnetic field B) Non-magnetic SPP LM under magnetic field C) F-MNP LM under NIR irradiation

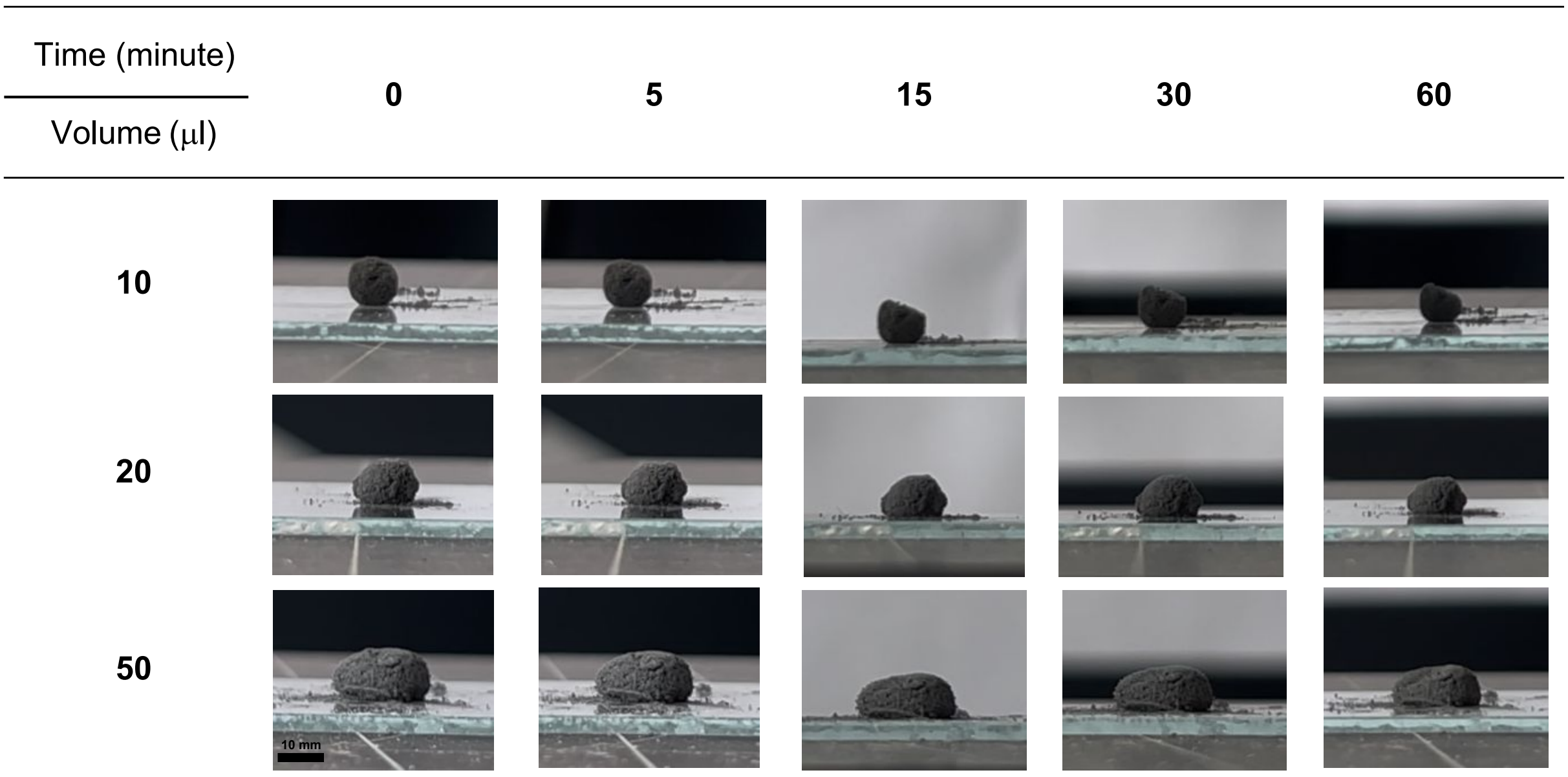

Figure S9. Investigation the stability of SPP FLM liquid marble in three different volumes

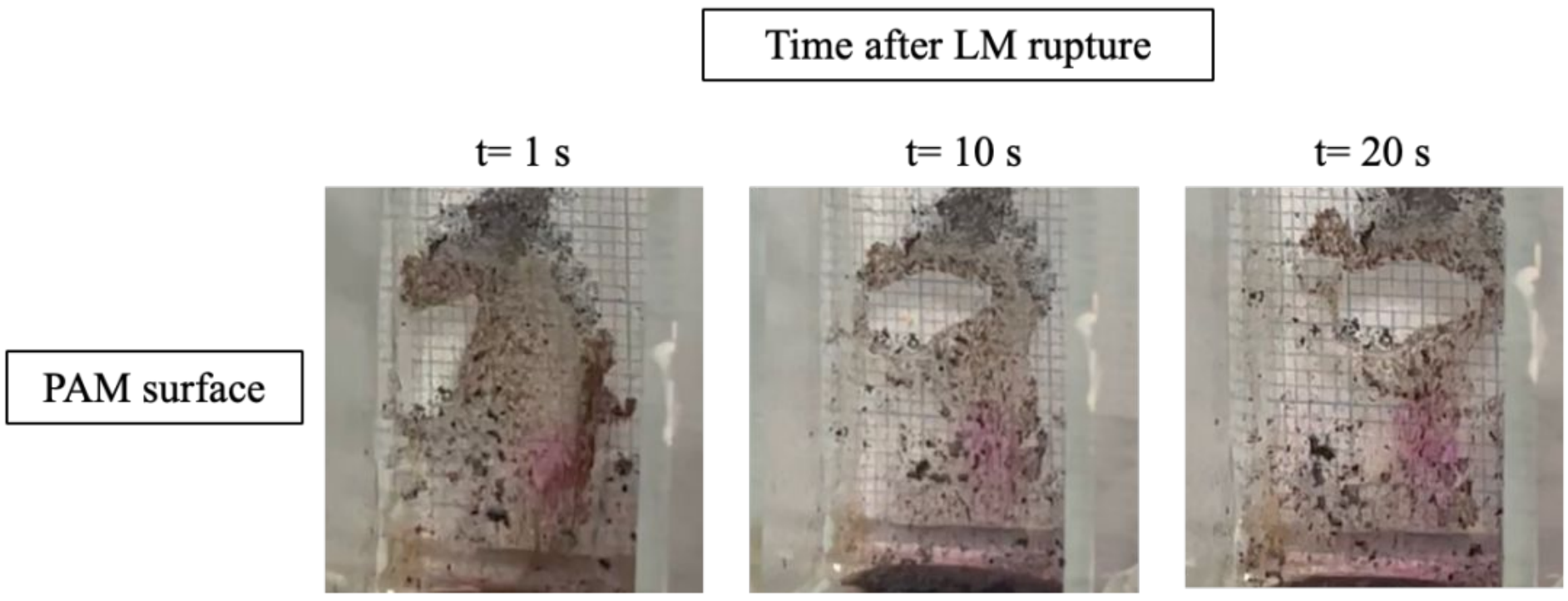

Figure S10. Sequential optical images illustrating localized pH-induced color change after liquid marble rupture on the PAM surface
